# High-Porosity Conjugated Polyelectrolytes Synthesized via Sonogashira–Hagihara Coupling in Concentrated Emulsions: Robust Adsorptive–Photocatalytic Hydrogels for Water Pollutant Removal

**DOI:** 10.1021/acs.macromol.5c02304

**Published:** 2026-01-06

**Authors:** Aleksander Saša Markovič, Siebe Lievens, Emeline Hanozin, Milica Velimirovic, Albin Pintar, Sebastijan Kovačič

**Affiliations:** † Catalysis and Organic Synthesis research group, Laboratory for Organic and Polymer Chemistry, Faculty of Chemistry and Chemical Engineering, 200917 University of Maribor, Smetanova 17, SI-2000 Maribor, Slovenia; ‡ Department of Inorganic Chemistry and Technology, National Institute of Chemistry, Hajdrihova 19, SI-1001 Ljubljana, Slovenia; § 54520 Flemisch Institute for Technological Research (VITO), Boeretang 200, 2400 Mol, Belgium

## Abstract

Conjugated polyelectrolyte (CPE) hydrogels uniquely combine π-conjugation, ionic functionality, and water compatibility in a single-polymer network. This work reports on the design, synthesis, and application of high-porosity CPE hydrogels obtained via the Sonogashira–Hagihara cross-coupling reaction as a polymerization chemistry in a high internal phase emulsion (HIPE) template. In this way, we combine the hydrophilic and π-conjugated electronic properties of CPEs with the high porosity of polymerized high internal phase emulsions (polyHIPEs or PHs), enabling the development of a multifunctional polymer platform. High-porosity CPE-PHs exhibit a surface area of up to 355 m^2^·g^–1^, excellent water uptakes of up to ∼25 g·g^–1^, and visible-light absorption with band edges at 720 and 610 nm and band gaps of 2.35 and 2.47 eV for anionic CPE-PH–SO_3_® and cationic CPE-PH-NMe_3_^+^, respectively. These CPE-PHs are then used to remove the endocrine-disrupting chemical bisphenol A (BPA) as a model water pollutant. The CPE-PH–SO_3_® demonstrates exceptional performance, achieving overall removal efficiencies of 93% and 96% through synergistic adsorption (∼71% and ∼50%, respectively) and visible light-driven photocatalysis (∼22% and ∼46%, respectively) during 8 and 24 h experiments. These efficiencies are among the highest reported for organic photocatalyst. In contrast, the cationic analogue CPE-PH-NMe_3_^+^ suffers from oxidative degradation and thus limited activity. Stability studies confirmed that CPE-PH–SO_3_® retains its structural and electronic integrity during prolonged operation. These results demonstrate the potential of high-porosity CPE-PH hydrogels as a multifunctional polymer platform that synergistically integrates adsorption and heterogeneous photocatalysis for robust and efficient water applications.

## Graphical abstract

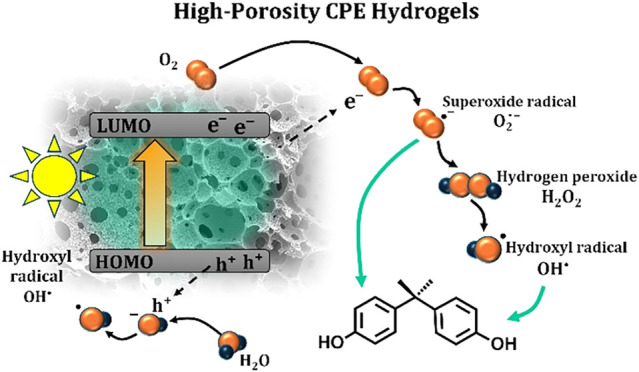

## 1 Introduction

Conjugated polyelectrolytes (CPEs) are a class of organic semiconductors that feature a π-conjugated backbone decorated with ionic side chains, combining electronic conductivity with solubility in polar solvents.^1–3^ Pendant ionic functionalities are of particular importance as they can improve ionic conductivity, enable water-processability, and promote hydrogel formation.^4–6^ When physically or chemically cross-linked and swollen with water, CPEs form hydrogels; soft, ion-conducting networks capable of storing large volumes of water while retaining the electronic properties of the conjugated framework. These CPE hydrogels have recently attracted attention due to their potential for a wide range of redox-active, light-driven, and catalytic processes, including water splitting, environmental remediation, photodynamic therapy, and enzymatic electron transfer.^1,7,8^ Despite their versatility, most CPE hydrogels have a dense structure that limits bulk accessibility and thus reduces the accessible reactive surface sites. To address this limitation, the focus here shifts to integrating porosity into CPE hydrogels, a strategy that is well established in the realm of conjugated porous polymers (CPPs).^9^

CPPs combine a π-conjugated electronic system with permanent porosity extending through a multidimensional (2D and 3D) polymer network, allowing the simultaneous transport of charges, ions, and molecular species. Their rigid, cross-linked network provides a large surface area, while the introduction of porosity improves accessibility, mass transport, and reactive surface area. These features make CPPs an attractive platform for energy storage, heterogeneous catalysis, environmental remediation, and sensing.^10–16^ A particularly interesting subclass of CPPs are those that exhibit hierarchical porosity, which significantly improves the diffusion of solutes and enables their three-dimensional distribution. A promising strategy to introduce such a hierarchy into the porous architecture is the templating of macroporosity through concentrated emulsions such as high internal phase emulsions (HIPEs),^17^ a powerful and versatile technique leading to polyHIPEs (PHs) after curing.^18^ The morphology of PHs depends on several formulation and processing factors, including the choice and ratio of porogenic solvents,^19^ controlled coarsening (shear or aging),^20^ the length and concentration of the block copolymer surfactant,^21^ and possible microphase separation during polymerization.^22^

Conjugated polyHIPEs are a distinct class of CPPs with a hierarchically structured 3D-interconnected and π-conjugated porous framework. The “first generation” of π-conjugated PHs were synthesized using transition-metal (TM)-catalyzed reactions such as Suzuki–Miyaura,^23^ Sonogashira–Hagihara,^8,24^ or chain-growth insertion polymerization.^25,26^ These reactions proved valuable in establishing a synthetic pathway for bringing functionality into the PH backbone by inserting arylene-vinylene or arylene-ethynylene building blocks, thereby creating semiconducting polymer networks. Following this “first generation”, Kotnik et al. started to develop TM-free synthetic routes by introducing Knoevenagel, Schiff-base, or Staudinger chemistry, which led to macroporous polymer networks based on arylene-cyano-vinylene,^27^ arylene-imine,^28^ arylene-azine,^29^ or arylene-iminophosphorane building blocks.^30^ Due to their structural features and the wide variety of π-conjugated building blocks available for their synthesis, these PHs have rapidly developed into a versatile platform that finds advantageous applications as photocatalysts for singlet oxygen generation,^23,31^ in organic photoredox reactions^24,32^ and as photoinitiators in radical polymerizations^33^ or photodegradation of organic pollutants in water.^8,26^ Despite the great potential of π-conjugated PHs, they have so far been synthesized exclusively from neutral, often hydrophobic building blocks, which limits their water compatibility and, thus, their use in aqueous media. Although no studies have yet reported the combination of polyelectrolyte functionality, π-conjugated electronic system, and PH morphology in a single-polymer network, bridging this gap is important given the growing demand for highly porous photocatalytic hydrogels that function efficiently in aqueous environments.

Until now, the preparation of photocatalytic hydrogels has often relied on a side-chain functionalization strategy, where organic chromophores (e.g., eosin Y, porphyrins, or perylenediimides) were immobilized on otherwise saturated hydrocarbon backbones.^34–36^ These polymer-supported photosensitizers enable modular synthesis, robust light harvesting, and have been widely used as heterogeneous photocatalysts capable of photochemically generating various reactive oxygen species for synthetic, biological, or environmental applications.^31,37–39^ In contrast to the state of the art, we propose a fully π-conjugated network integrated throughout a porous hydrogel framework.

This work presents HIPE-templated conjugated polyelectrolytes (CPE-PH) as a new porous photocatalytic hydrogel platform that combines electronic conductivity, ionic functionality, water compatibility, and high porosity within a single framework. The poly­(arylene-ethynylene) skeleton was constructed through a Sonogashira–Hagihara condensation reaction between the substituted dibromohydroquinone monomers, i.e., 2,5-bis­(3-[*N*,*N*,*N*-trimethylamino]-1-oxapropyl)-1,4-dibromobenzene (M-NMe_3_^+^) and 1,4-dibromo-2,5-bis­(3-sulfonatopropoxy)­benzene disodium salt (M-SO_3_®), and an aromatic 1,3,5-triethynylbenzene (TEB). The resulting CPE-PH hydrogels thus contain covalently bound trimethylamine cations (-NMe_3_^+^) and sulfonate anions (−SO_3_®) as side chains on the conjugated poly­(arylene-ethynylene) backbone. Considering the π-conjugated backbone with hydrophilic ionic side chains, the obtained CPE-PHs allow good water absorption, while their extended π-conjugated electronic system confers visible-light activity. Therefore, we investigated the ability of these CPE-PH hydrogels to remove the model pollutant bisphenol A (BPA) from water, as one of the most well-known and prevalent endocrine-disrupting chemicals in ground and surface water. We discovered that CPE-PH hydrogels operate with dual-function, i.e., a combination of adsorption and visible light-driven heterogeneous photocatalysis.

## 2 Experimental Section

Supporting Information contains a comprehensive list of chemicals. Descriptions of analytical techniques (i.e., NMR spectroscopy, FTIR spectroscopy, SEM microscopy, nitrogen physisorption, He-pycnometry, UV–vis DR spectrophotometry, cyclic voltammetry, DART-MS, and LC-Q-ToF) used to characterize CPE-PHs, photocatalytic activity of the prepared polymer network, and degradation products are detailed. Synthesis and detailed results of the characterization of the monomers, polymers, and foams used in this work are also presented.

### 2.1 Synthesis of Ionic Aryl Dibromide Monomers (M-NMe_3_^+^ and M-SO_3_®)

1 mmol of 2,5-dibromohydroquinone, 2.5 mmol of (3-bromopropyl)­trimethylammonium bromide or 1,3-propane sultone, and 1.25 mmol of NaOH were dissolved in 4 mL of ethanol. The reaction mixture was refluxed at 80 °C. After cooling to room temperature, the product was collected by filtration. A more detailed description can be found in the Supporting Information.

### 2.2 Synthesis of CPE polyHIPEs

0.035 mmol of Pd­(PPh_3_)_4_ (0.035 mmol) was added to a vial, 10 wt % pluronic F-108, 0.35 mmol of monomer, an excess of 1,3,5-triethynyl benzene (1.5 equiv), and 2.1 mmol of DABCO were dissolved in 1.5 mL of DMSO. To this mixture, 4.5 mL of petroleum benzine was added dropwise as internal phase. The emulsion was transferred and polymerized overnight at 80 °C. The resulting monolith was first purified by Soxhlet extraction with a 1:1 ethanol–water mixture for 24 h. The remaining impurities were then removed by supercritical CO_2_ drying to obtain a macroporous polymer foam. A more detailed description can be found in the Supporting Information.

### 2.3 Photodegradation Tests

A batch reactor was covered with aluminum foil, and all ambient lights were turned off, then 250 mL of 10 ppm BPA solution and 32.5 mg of an appropriate catalyst were added. The solution was aerated at 750 mL/min and two aliquots were taken during the dark phase (2 h); after 2 h, the light with a cutoff filter at 420 nm was turned on and aliquots were taken at different periods, i.e., 1, 2, 3, 4, 5, and 6 h during the light phase (for 8 h experiment). The samples were stored in a refrigerator to keep them from degrading further, until they were prepared for further analysis. For recyclability tests, after each run, the monolith was removed, rinsed with water and acetone, gently dried at 60 °C for 2 h, and reused under identical conditions.

### 2.4 Detection of the BPA Degradation and Intermediates

To confirm and map the presence of Bisphenol A (BPA) and its transformation products generated during photocatalysis, a detailed analysis was performed using both Direct Analysis in Real Time (DART) mass spectrometry and liquid chromatography coupled with quadrupole time-of-flight mass spectrometry (LC-Q-ToF). DART ionization source (IonSense Inc., Saugus, MA, USA) was coupled with a Q Exactive Orbitrap high-resolution mass spectrometer (Thermo Fisher Scientific, Bremen, Germany). LC-Q-ToF measurements were performed on a timsTOF Pro2 (Bruker Daltonics, Bremen, Germany) equipped with an electrospray ionization source. The chromatographic system consisted of an Elute+ UHPLC pump (HPG1300, Bruker Daltonics, Germany) equipped with a C18-BEH-phenyls analytical column (2.1 mm × 100 mm, 1.7 μm; Waters Corporation, USA), maintained at 40 °C. The injection volume was 5 μL, and the flow rate was 0.40 mL/min. The mobile phase consisted of solvent A, ultrapure water (Chromasolv LC-MS Ultra, Honeywell Chemicals, USA) containing 2 mmol/L ammonium acetate (≥98%, VWR Chemicals, Belgium), and solvent B, acetonitrile (LC-MS grade, Biosolve B.V., Netherlands). The gradient elution program began at 80% A, linearly decreased to 5% A over 8 min, held at 5% A for 2 min, then returned to 80% A in 0.10 min, and was maintained for 1.90 min to re-equilibrate the column. In both systems, measurements were performed in negative ionization mode.

## 3 Results and Discussion

### 3.1 Monomer Synthesis

The synthesis of the ionic building blocks, i.e., 2,5-bis­(3-[*N*,*N*,*N*-trimethylamino]-1-oxapropyl)-1,4-dibromobenzene (M-NMe_3_^+^) and 1,4-dibromo-2,5-bis­(3-sulfonatopropoxy)­benzene disodium salt (M-SO_3_®), is shown in Figure 1a. Briefly, dibromohydroquinone **(1)** reacted with (3-bromopropyl)­trimethylammonium bromide **(2)** or 1,3-propane sultone **(3)** in refluxing ethanol in the presence of excess sodium hydroxide for 48 h. The nonoptimized total yields of M-NMe_3_^+^ and M-SO_3_® were 95.5% (600 mg) and 99.5% (1110 mg), respectively. The molecular structures of M-NEt_3_^+^ and M-SO_3_® were further elucidated by ^1^H and ^13^C NMR spectroscopy (Figures S1, S2, S3, and S4). The presence of ionizable side groups, propyltrimethylammonium and propylsulfone, was confirmed by the appearance of the following protons. Methylene protons **b** (quintet) at δ 2.28 and 2.24 ppm for M-NMe_3_^+^ and M-SO_3_®, respectively, the methylene protons **a** (triplets) near to the oxybenzene at δ 4.12 and 4.05 ppm, and methylene protons **c** (triplets) by the CH_2_–SO_3_^®^ at δ 3.12 and CH_2_–NMe^+^ at δ 3.62, respectively. In addition to a singlet signal at δ 3.05 ppm, which is due to the CH_3_ protons of the M-NMe_3_^+^ in both monomers, we see the aromatic protons at δ 7.34 and 7.30 ppm. M-NMe_3_^+^ and M-SO_3_® were then further used for the synthesis of the hydrophilic π-conjugated PHs based on the CPE-NMe_3_^+^ **(5)** and CPE-SO_3_® **(6)** networks (Figure 1b).

**Figure 1:**
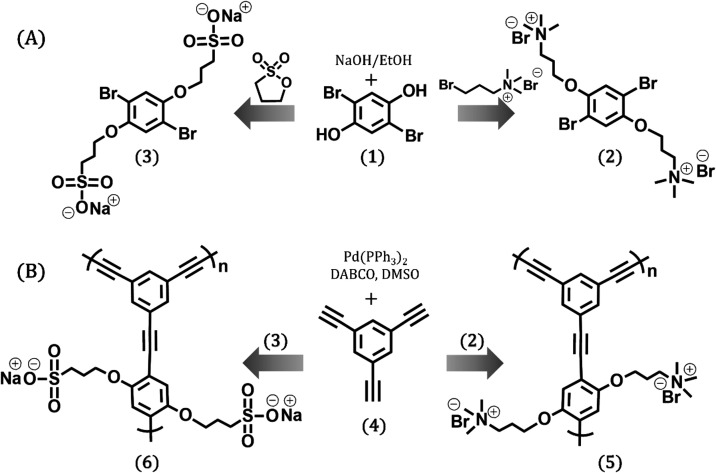
(A) Synthesis of 2,5-bis­(3-[*N*,*N*,*N*-trimethylamino]-1-oxapropyl)-1,4-dibromobenzene (M-NMe_3_^+^) (2) and 1,4-dibromo-2,5-bis­(3-sulfonatopropoxy)­benzene disodium salt (M-SO_3_®) (3) monomers and (B) ethynylene-linked CPE networks (CPE-NEt_3_^+^) (5) and (CPE-SO_3_®) (6).

### 3.2 CPE Network Synthesis and Optimization of Polymerization Conditions

Prior to the synthesis of CPE-NMe_3_^+^- and CPE-SO_3_®-based PHs through Pd-catalyzed Sonogashira–Hagihara coupling, the reaction profile was optimized to be compatible with the subsequent two-phase HIPE system. Therefore, a model reaction between ionically charged M-NMe_3_^+^ **(2)** or M-SO_3_® **(3)** and apolar triethynylbenzene (TEB) **(4)** was performed in which different experimental variables such as solvent, catalyst system, amine base, and temperature were examined. The first step was finding a suitable solvent that could dissolve all the reagents (Table S1). This proved to be difficult as the mixture of monomers (M-NMe_3_^+^, M-SO_3_®, and TEB), the base (NEt_3_ or DABCO), and a catalyst (Pd^0^/Pd^II^/Cu^I^) did not result in a homogeneous solution. A solvent screen revealed ethylene glycol (EG), a combination of *N*-methylpyrrolidone (NMP)/H_2_O and DMSO as the most appropriate for the preferred system in this study. A Sonogashira–Hagihara coupling reaction was then carried out in EG, NMP/H_2_O, or DMSO and catalyzed with Pd­(OAc)_2_ at 80 °C. Surprisingly, the yields obtained in EG or NMP/H_2_O were over 100% (despite the optimized workup procedure; see the SI), whereas the reactions carried out in DMSO resulted in yields of 27 and 43% for P-NMe_3_^+^ and P-SO_3_®, respectively. Higher yields in the case of EG or NMP/H_2_O were probably the result of the side-reaction, i.e., oxo-addition of the solvent to alkyne groups in TEB.^40^ To investigate possible side-reactions, a simple benchmark reaction was performed with phenylacetylene and EG or NMP/H_2_O, which was assessed using ^1^H NMR (Figure S5). An oxo-addition of the solvent to the alkyne group in phenylacetylene occurred (Figure S6), as evidenced by the disappearance of the alkyne-proton resonance at 3 ppm and the appearance of a new broad signal at 7.45 ppm, which is assigned to the formation of polyacetylene.

Next, we investigated the 1,4-diazabicyclo[2.2.2]­octane (DABCO) as a substitute for the triethylamine (Et_3_N) base. While Et_3_N worked well in the above model reactions, its boiling point is close to 80 °C at which the Sonogashira–Hagihara reaction was performed and consequently began to boil during polymerization, resulting in large bubbles in the final CPE monoliths. The boiling point of Et_3_N is likely to be very unfavorable for the stability of the latter HIPE formulation. Therefore, the solid amine base DABCO with a boiling point of 174 °C instead of Et_3_N was used. In the reaction catalyzed by DABCO (p*K*_a_ ≈ 8.9 in DMSO), the gel point was reached after 1 h, forming a solid, homogeneous gel, whereas in the reaction catalyzed by Et_3_N (p*K*_a_ ≈ 9.0 in DMSO), the gel point was reached after about 2 h, with yields of 77 and 82% for P-NMe_3_^+^ and P-SO_3_®, respectively.

Finally, the catalytic system was thoroughly assessed. Pd­(OAc)_2_ was effective but resulted in relatively low yields of 27 and 43% for P-SO_3_® and P-NMe_3_^+^, respectively. Therefore, the Pd­(OAc)_2_/CuI catalyst system was replaced by the Pd­(PPh_3_)_4_/CuI system. This change led to improved yields of 62 and 63% for P-SO_3_® and P-NMe_3_^+^, respectively. Although the addition of the copper­(I) halide increases the reactivity of the system, it also favors the undesired alkyne homocoupling through a Hay/Glaser reaction in the presence of oxygen.^41^ Since oxygen cannot be avoided in the subsequent HIPE polymerization, another benchmark experiment was carried out, in which only Pd­(PPh_3_)_4_ as the catalyst (no CuI) and TEB were dissolved in DMSO. The reaction was carried out at 40 °C in an air atmosphere. Surprisingly, the TEB solution stopped flowing after about an hour when the gel point was reached, indicating that the ethynyl groups of TEB reacted and formed a network. This proves that alkyne homocoupling occurs even when CuI is not present as a cocatalyst, so it cannot be avoided in the subsequent HIPE polymerization.

### 3.3 Structuring the High Porosity into CPE Hydrogels

As shown in Table S2, the Sonogashira–Hagihara coupling of ionically charged M-NMe_3_^+^ or M-SO_3_® with apolar TEB at 80 °C under air conditions in DMSO catalyzed by Pd­(PPh_3_)_4_ could be carried out without the use of CuI, resulting in faster reaction time, good yields, and fewer side reactions. This optimized reaction profile was then applied as the polymerization chemistry in a two-phase HIPE formulation. In HIPE, DMSO was used as the continuous phase, as it dissolves all monomers and catalysts, while petroleum benzine served as the internal phase. These two immiscible liquids were stabilized by a polymeric surfactant (Pluronic F-108), resulting in oil-in-oil (O/O) HIPEs.^17^ The O/O HIPE polymerization via Sonogashira–Hagihara coupling was carried out at 80 °C in an air atmosphere and catalyzed with the combination Pd­(PPh_3_)_4_/DABCO. This catalyst system proved to be very efficient and ensured rapid gelation of the HIPE system. After 24 h reaction time, it was confirmed that all monomers had been consumed. The obtained CPE-PH-NMe_3_^+^ and CPE-PH–SO_3_® were purified by Soxhlet extraction for 1 day using EtOH, followed by scCO_2_ drying. The overall polymerization yields, given as the mass ratio of the purified monoliths to the theoretical masses assuming 100% M-SO_3_® and M-NMe_3_^+^ conversion, were 81 and 64% for CPE-PH-NMe_3_^+^ and CPE-PH–SO_3_®, respectively, indicating successful removal of all nonpolymerizable components as well as soluble oligomeric chains.

The PH densities (ρ_PH_) were 0.056 and 0.06 g·cm^–3^ for the CPE-PH–SO_3_® and CPE-PH-NMe_3_^+^, respectively, and the total porosity of the PHs was about 97%, calculated from ρ_PH_ and the density of the polymer (ρ_P_) at about 1.7 g·cm^–3^ (based on He-pycnometry measurements of the skeletal densities; Table S4). The porous structures of the dried CPE-PHs were characterized by using standard scanning electron microscopy (SEM). The CPE-PHs exhibited highly interconnected, open-cell, porous structures typical of polyHIPEs, as shown in Figure 2 for CPE-PH–SO_3_® and CPE-PH-NMe_3_^+^, respectively. The void diameters, *d*_v_, are in the range of 25–35 μm, and the interconnecting window diameters, *d*_w_, range between 5 and 10 μm.

The porous properties and associated specific surface areas (*S*_BET_) were further analyzed by using nitrogen adsorption–desorption measurements. The nitrogen physisorption reveals a typical type II isotherm (Figure 2c) and by applying the Brunauer–Emmett–Teller (B.E.T.) theory to the nitrogen adsorption data within the 0.06–0.30 *p*/*p* ^0^ range, a specific surface area (SSA) of up to 353 m^2^·g^–1^ (sample CPE-PH–SO_3_®) or 299 m^2^·g^–1^ (sample CPE-PH-NMe_3_^+^). The increase in nitrogen uptake in regions below 0.1 and above 0.5 *p*/*p* ^0^ indicates a hierarchically porous material consisting of micro- and macropores. In general, standard PHs have a rather low SSA (usually between 10 and 20 m^2^·g^–1^) because of their inherent macroporosity and a negligible amount of micro/mesoporosity. Additional treatments such as hyper-cross-linking^42,43^ or the addition of an organic porogen to the monomer phase^44^ can increase *S*_BET_. In our case, however, we believe that a microsyneresis process^45^ is responsible for the increase of *S*_BET_ in both samples, CPE-PH–SO_3_® and CPE-PH-NMe_3_^+^, with the porogenic solvent being a key. In practice, this solvent was DMSO, which played a dual role in our system. On the one hand, it is a continuous phase of the HIPE template, but second, it also serves as a porogen in the form of nanodroplets around which the polymer network grows. The process of microsyneretic morphology formation (Figure 2d) within the void walls of PH proceeds as follows. As a highly cross-linked network is formed through the cross-coupling of the three ethynyl groups of TEB with M-SO_3_® and M-NMe_3_^+^ in the polymer chains, the polymer network is increasingly compressed and eventually expels DMSO nanodroplets from the formed polymer gel. This leaves micropores in the void walls after the PH is dried, the volume of which was responsible for the higher *S*_BET_.

**Figure 2:**
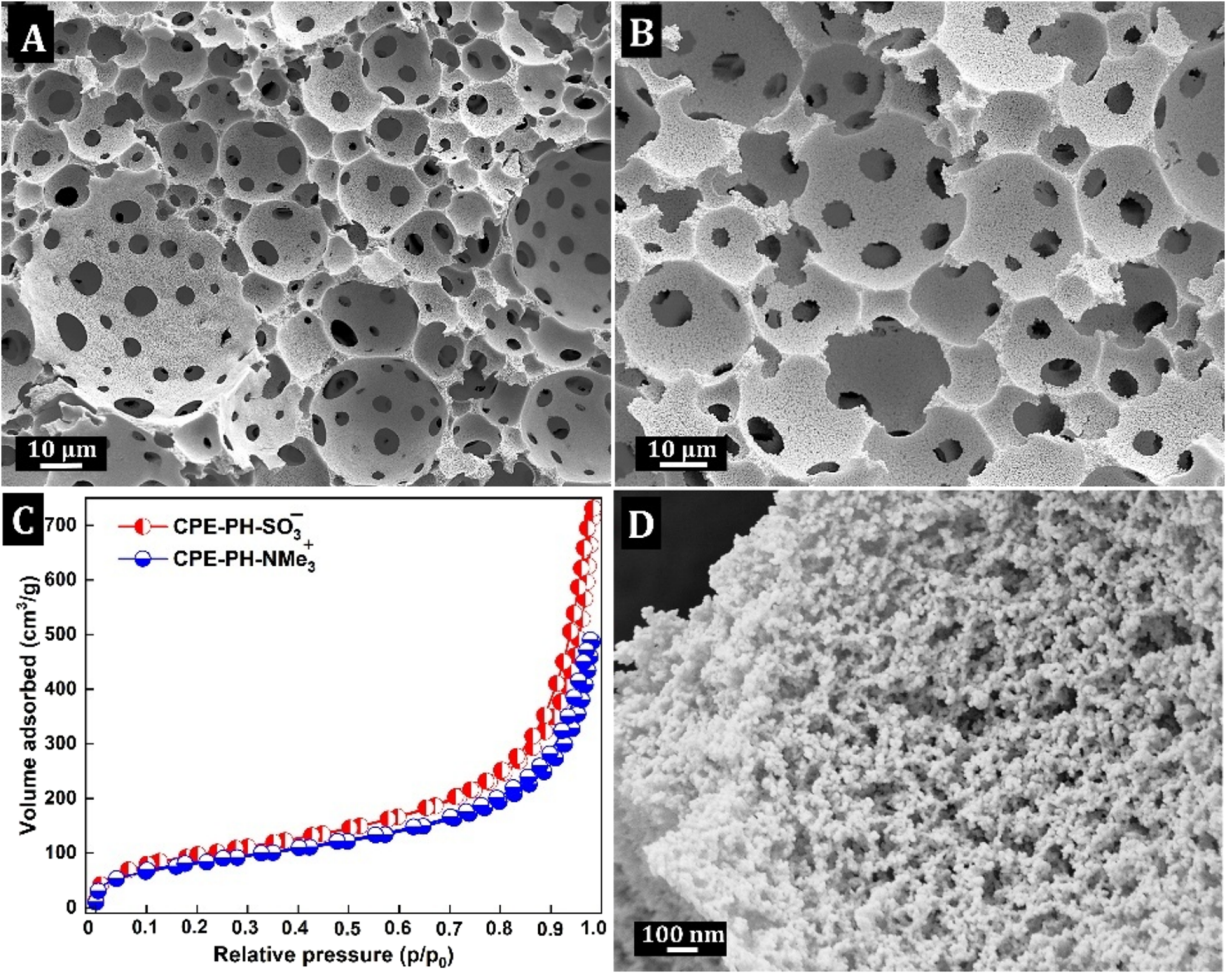
SEM image of CPE-PH–SO_3_® (A) and CPE-PH-NMe_3_^+^ (B), N_2_ sorption isotherms (C), and high-magnification SEM images of void walls in CPE-PH-NMe_3_^+^(D).

The chemical characterization of the CPE-PH included elemental analysis (EA), Fourier transform IR (FTIR), diffuse reflectance UV/vis (DR UV/vis) spectroscopy, and solid-state NMR spectroscopy (^13^C CP/MAS NMR). Examination of the elemental composition of the CPE-PH–SO_3_® and CPE-PH-NMe_3_^+^ samples by EA confirmed the inclusion of M-SO_3_® and M-NMe_3_^+^ in the macromolecular network, as shown by the presence of nitrogen (N) and sulfur (S) atoms. The EA data revealed C 55.9%, H 5.2%, N 2.9%, S 3.0%, and O 33% or C 65.0%, H 5.6%, N 3.7%, and O 25.7% for CPE-PH–SO_3_® and CPE-PH-NMe_3_^+^, respectively (Table S3). The FTIR spectra of CPE-PH–SO_3_® and CPE-PH-NMe_3_^+^ are shown in Figure S7. The bands common to CPE-PH-NMe_3_^+^ include those at 3680 cm^–1^ (quarter ammonium salts), 1120 cm^–1^ (C–N stretching), and bands at 1480 and 960 cm^–1^ correspond to the CH_3_ stretching and bending vibrations, respectively, of the NMe_3_^+^ group in the CPE-PH-NMe_3_^+^. On the other hand, typical bands for the CPE-PH–SO_3_® network include those at 1220 and 1039 cm^–1^, corresponding to the asymmetric and symmetric S–O stretching vibrations of the SO_3_® group, respectively. Both samples show bands at 2208 cm^–1^ (−C≡C– stretching) and 1582 cm^–1^ (−C_Ar_=C_Ar_– stretching). The ^13^C CP/MAS NMR spectra (Figure S8) of the CPE-PH–SO_3_® and CPE-PH-NMe_3_^+^ samples show a broad, partially resolved signal in the δ 120–145 ppm range corresponding to the aromatic carbons (−C_Ar_=C_Ar_−) and the signals at about δ 90 and 80 ppm are due to the ethynyl carbons (−C≡C−). The incorporation of ionic side chains into the PAE backbone is indicated by the characteristic signal at δ 70 ppm (C_Ar_–OCH_2_−). The signals at δ 45 and 55 ppm are due to the CH_3_ carbons in the NMe_3_^+^ group and the CH_2_ carbon adjacent to the NMe_3_^+^ group, respectively, in CPE-PH-NMe_3_^+^. The signal at δ 45 ppm in CPE-PH–SO_3_® is due to the CH_2_ carbon adjacent to the SO_3_® group, while the signal at δ 55 ppm is assigned to DABCO-H^+^ (labeled with an asterisk),^46^ as a counterion for the sulfonic group. The presence of DABCO in CPE-PH–SO_3_® is somehow surprising, but EA shows an N content of 2.9% for CPE-PH–SO_3_®, where no N should be present. In both samples, there is also a signal at about δ 20 ppm, corresponding to the middle CH_2_ carbon in the side chain.

To further support the high degree of condensation of the network, we quantified the chemical gel fraction under harsh extraction. Model CPE-PH cubes were refluxed in DMSO at 150 °C for 24 h and then rinsed, dried, and reweighed. The resulting gel content was approximately 92.5%, confirming that most of the material is an insoluble, permanently cross-linked network rather than a partially condensed gel. This is consistent with the spectroscopic characterization of an arylene–ethynylene backbone (^13^C CP/MAS NMR, FTIR), the retention of monolithic form after Soxhlet/scCO_2_ processing, and the persistent high porosity and surface area characteristic of HIPE-templated networks. Together, these orthogonal characterizations confirm the successful formation of a highly cross-linked CPE-PH network.

### 3.4 Bisphenol A (BPA) Removal by Synergetic Adsorption–Photocatalysis

The photocatalytic activities of CPE-PHs were evaluated for BPA removal under simulated visible-light (Vis) irradiation and compared with poly­(arylene-ethynylene) (PAE)-PH as a neutral nonionic control. To distinguish the contribution of direct photolysis or adsorption in the photocatalytic treatment of BPA, two control tests were performed using either visible-light irradiation without photocatalyst in the aqueous solution or a BPA/photocatalyst suspension without light irradiation (dark phase) (Figure 3). In the absence of the photocatalyst, BPA degradation during the 8 h simulated visible-light-driven experiment was extremely low (<0.5% change in BPA concentration). This confirms that the contribution of BPA degradation via a direct Vis-light photolysis can be neglected. The second control was the adsorption test, i.e., during the dark phase of the applied photocatalytic treatment of BPA, in which adsorption equilibrium is reached within 120 min (Figure 3d).

**Figure 3:**
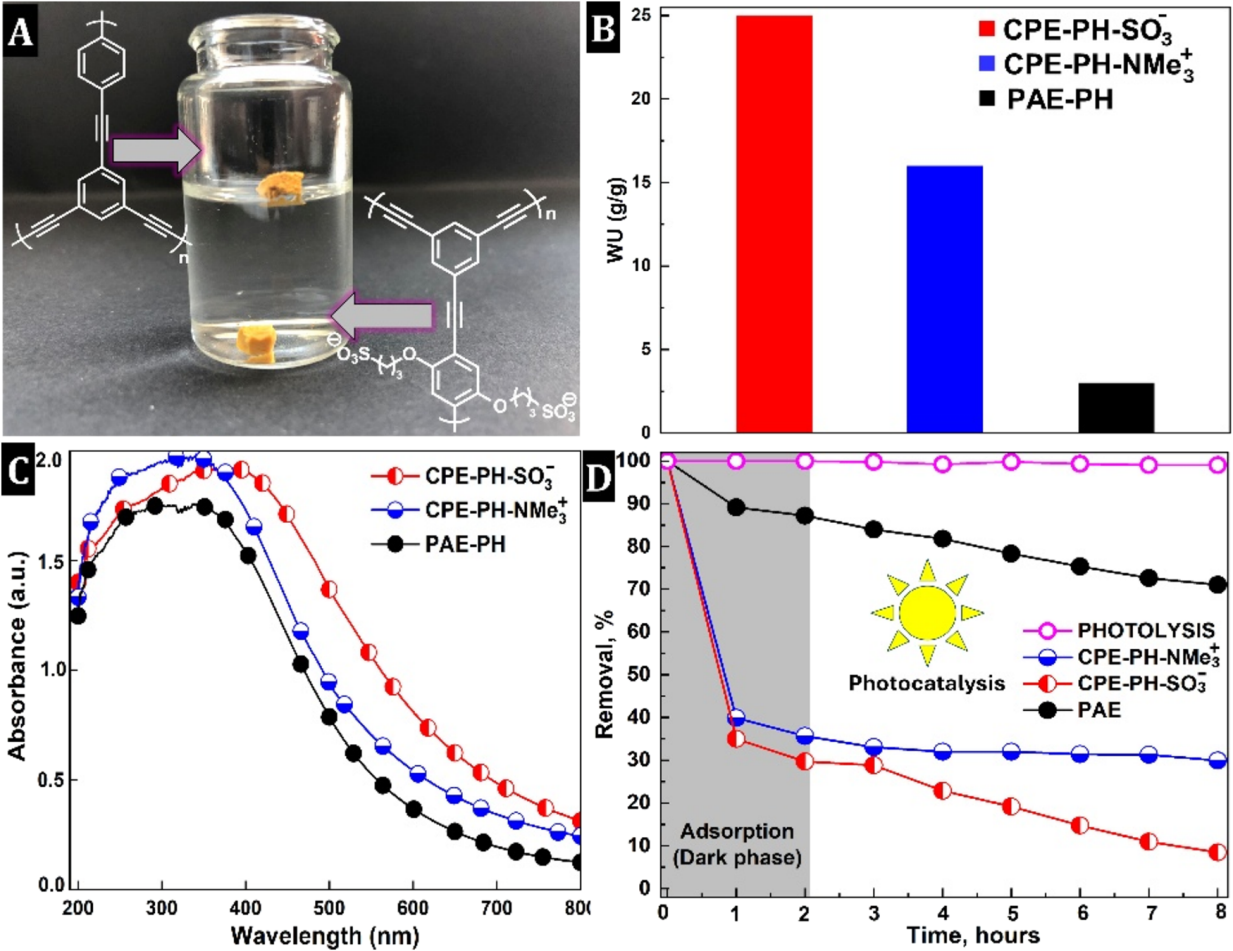
Photo of PAE–PH and CPE-PH–SO_3_® monoliths sink in water (A); water uptake (B); UV–vis DRS analysis of the PHs (C); and adsorption/visible-light-driven photooxidation of water-dissolved BPA (D).

#### 3.4.1 Adsorption Performance of CPE-PHs

The BPA adsorption for CPE-PH–SO_3_®, CPE-PH-NMe_3_^+^, and PAE–PH is 71, 65 and 12%, respectively (Figure 3d). Obviously, both CPE-PHs strongly adsorb BPA, with the CPE-PH–SO_3_® outperforming the CPE-PH-NMe_3_^+^, while the neutral PAE–PH hardly exhibits any adsorption. Apparently, the side chains with ionic functionality are key. They confer water-compatibility to the inherently hydrophobic π-conjugated macromolecular network, giving CPE-PHs good water uptake (*W*_U_) properties. They can absorb up to 25 g·g^–1^ of water (PAE–PH absorbs only 3 g·g^–1^; see Table S5 and Figure 3b) and can sink in water (PAE–PH floats on the surface; Figure 3a). Based on these facts, the high BPA adsorption could be rationalized as follows. The water uptake is followed by swelling of the CPE-PH backbone (swelling ratio of 1.5), leading to expansion of the π-conjugated macromolecular network, making it highly accessible for interaction with BPA. This swelling-driven network expansion is therefore the probable reason for the high adsorption capacity of CPE-PH. However, there are at least two different mechanisms that drive adsorption. First, through the π–π electron donor–acceptor interaction between TEB knots with the macromolecular network and BPA,^26,47,48^ the phenomenon in which a π–π electron coupling occurs between the electron-poor regions (considered as π-acceptors), in this case the benzene rings of BPA, and the electron-rich regions, in this case the TEB within the network (considered as π-donors). Second, the side chains (especially the sulfonates) help to capture BPA through H-bonding.

#### 3.4.2 Photocatalytic Activity of CPE-PHs for BPA Removal

The ability of CPE-PH to absorb visible light, combined with its good water absorption and high specific surface area, prompted us to investigate these networks as a heterogeneous photocatalyst for the visible light-driven photooxidation of BPA dissolved in water and to compare them with nonionic PAE–PH. First, UV–vis diffuse reflectance spectroscopy was used to study the light-harvesting ability and revealed that both CPE-PH–SO_3_® and CPE-PH-NMe_3_^+^ are visible-light-active materials (Figure 3c).

The UV–vis DRS spectra display a broad band with a maximum at around 387, 348, and 323 nm for CPE-PH–SO_3_®, CPE-PH-NMe_3_^+^, and PAE–PH associated with the π → π* transition, and corresponding optical absorption band edge at around 720, 610, and 595 nm for CPE-PH–SO_3_®, CPE-PH-NMe_3_^+^, and PAE–PH (Figure 3c). The band gap of CPE-PH–SO_3_® and CPE-PH-NMe_3_^+^ was further determined through Kubelka–Munk transformed reflectance spectra. The Tauc plots revealed the optical band gaps of 2.35 and 2.47 eV, respectively.

Next, PHs were investigated as heterogeneous photocatalysts in the visible light-driven photooxidation of BPA dissolved in water. A typical photocatalysis experiment was performed with the dark/adsorption phase for 120 min followed by the photocatalysis phase, in which the reaction system was illuminated with visible light (λ > 420 nm) for an additional 6 h. As shown in Figure 3d, CPE-PH–SO_3_® revealed a BPA photodegradation removal of 22%, while CPE-PH-NMe_3_^+^ and PAE–PH degraded about 5 and 15% of BPA, respectively, indicating that the actual photocatalytic performance of CPE-PH–SO_3_® is the highest. Through a synergistic combination of strong BPA adsorption (∼71%) and efficient photocatalytic degradation (∼22%), it achieves an overall efficiency of about 93% removal. In contrast, CPE-PH-NMe_3_^+^ showed relatively high BPA adsorption (∼65%) but the lowest photocatalytic activity (∼5%) and an overall BPA removal of about 70%. The neutral PAE–PH exhibited poor adsorption (∼12%) and moderate photocatalytic activity (∼15%), resulting in the lowest overall removal of BPA (∼27%).

Given the superior photocatalytic performance of CPE-PH–SO_3_® compared to its cationic analogue CPE-PH-NMe_3_^+^, the anionic CPE-PH was selected for further mechanistic studies to elucidate the intermediates formation. Based on our previous finding that nonionic PAE–PH can generate reactive oxygen species (ROS) such as hydroxyl radicals (OH^•^) and superoxide anion radicals O_2_^•^ ® under visible-light irradiation,^8^ the fluorescent probe technique was first employed to determine the specific ROS generated by CPE-PH–SO_3_^–^ during photocatalytic action.

The coumarin (COUM)^49^ and the radical cation 2,2‘-azinobis­(3-ethylbenzothiazoline-6-sulfonate) (ABTS^•+^)^50^ were used as probes to determine whether the CPE-PH–SO_3_® can form OH^•^ and/or superoxide O_2_^•^ ® radicals under visible-light irradiation. Both tests were positive; coumarin turned into a strongly fluorescent 7-hydroxycoumarin (characteristic absorbance at 450 nm; Figure S9) by reaction with an OH^•^ radical (Figures S10 and S11), while the blue/green ABTS^•+^ chromophore (characteristic absorbance at 734 nm; Figure S12) decolorized through the reaction with photoinduced e^–^ or with O_2_^•^ ® (Figure S13). As shown in Figure 4, CPE-PH–SO_3_® exhibited HOMO and LUMO positions at +1.49 V and −0.87 V, respectively, and the reduction potentials of some active ROS species are within the CPE-PH–SO_3_® band gap (2.35 V). The pathways for the generation of active ROS are as follows (Figure 4). It is very likely that the photoinduced electrons (e^–^) at the CPE-PH–SO_3_® surface reduce O_2_, producing O_2_^•^ ® (−0.16 V).^51^ On the other hand, the photoinduced holes (h^+^) are not able to oxidize H_2_O directly to OH^•^ radicals, as the reduction potential of H_2_O/OH^•^ (+2.33 V)^51^ lies outside the band gap of the CPE-PH–SO_3_® (Figure 4). However, the COUM test was positive, so OH^•^ radicals should have been formed. Since the reduction potentials of O_2_^•^ ® /H_2_O_2_ (+0.94 V) and H_2_O_2_/OH^•^ (+0.38 V)^51^ are both within the CPE-PH–SO_3_® band gap, OH^•^ radicals are probably generated by the catalytic disproportionation reaction of H_2_O_2_, which is formed from O_2_^•^ ® via the hydroperoxyl radical (HO_2_^•^) (Figure 4).^52^

**Figure 4:**
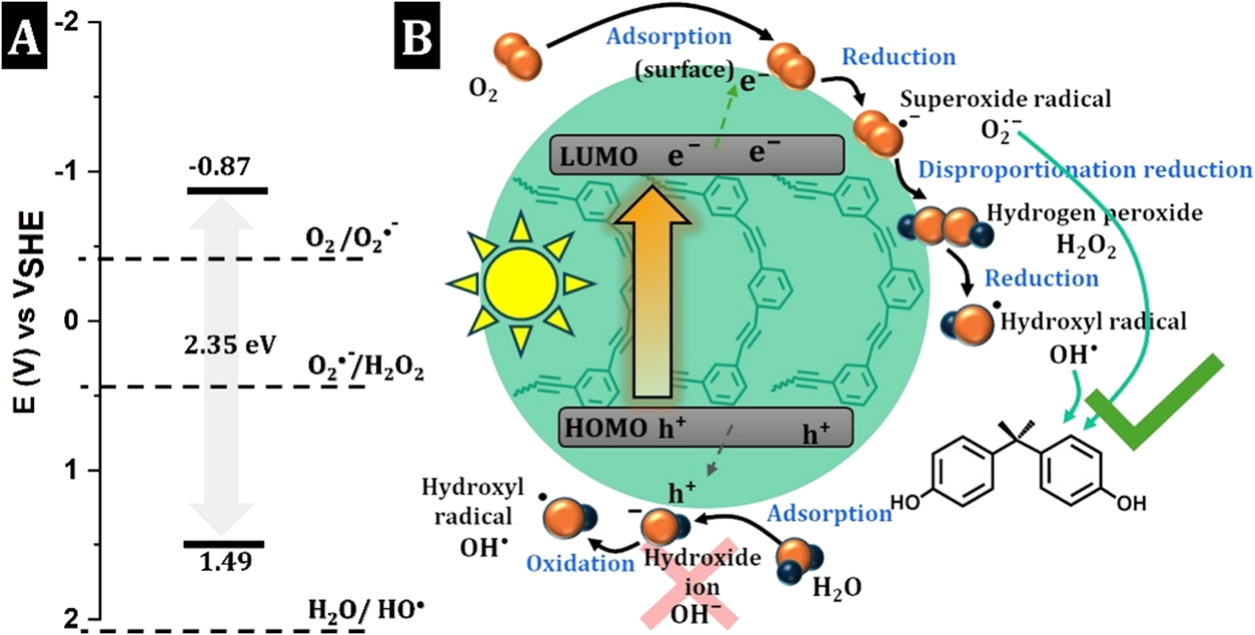
Molecular orbitals of CPE-PH–SO_3_® together with ROS redox potentials (A), proposed ROS-generation mechanisms (B).

#### 3.4.3 Photocatalytic Degradation Pathways and Intermediates Identification

A 24 h photodegradation experiment (2 h adsorption +22 h visible-light irradiation) was conducted to evaluate the photodegradation profile of BPA, the long-term photocatalytic performance and stability of the CPE-PH–SO_3_®. During the initial dark phase (2 h), over 50% of BPA was removed from the aqueous solution by adsorption (Figure 5a). After inducing visible light, a clear and immediate decrease in BPA concentration was further observed, indicating efficient photooxidation/photodegradation as an additional 46% of BPA was eliminated, achieving an overall removal efficiency of approximately 96% (Figure 5a). A detailed analysis of the degradation intermediates was performed by DART-MS and LC-ToF-MS, which confirmed not only the cleavage of the central isopropylidene bridge of BPA, but also a more extensive transformation of the aromatic system. Based on the identified photodegradation intermediates (Figure 5a), the phototransformation pathways of BPA in CPE-PH–SO_3_® solution are proposed as shown in Figure 5b, which are known intermediates in the mineralization process of BPA.^53,54^ The products with *m*/*z* 243.1035 (C_15_H_16_O_3_), 259.0984 (C_15_H_16_O_4_), and 133.0671 (C_9_H_10_O) are derived from the hydroxylation, oxidation, and C–C cleavage (β-scission) of C_15_H_16_O_2_ with *m*/*z* 227.1086 (BPA). Hydroxylation and combined oxidation/C–C cleavage have been described as the first steps of BPA phototransformation; therefore, monohydroxylated BPA (*m*/*z* 243.1035), isopropenylphenol (*m*/*z* 133.0671), and oxidized dihydroxylated BPA (*m*/*z* 259.0984) are the most frequently detected degradation intermediates. These intermediates are then further degraded through dealkylation, decarboxylation, or ring-opening to form compounds such as C_5_H_6_O_3_ (*m*/*z* 113.0257), C_8_H_8_O_3_ (*m*/*z* 151.0414), and C_9_H_12_O_2_ (*m*/*z* 151.0751) (Figure 5b). In particular, a C_15_H_16_O_5_ (*m*/*z* = 275.0936) fragment with five oxygen atoms was detected in larger quantities, suggesting an oxidative cleavage of the benzene ring and the formation of dicarboxylic acid species (Figure 5b). This intermediate is further degraded, probably by decarboxylation and ring fragmentation, to smaller species such as a C_5_ fragment (C_5_H_6_O_3_ with a *m*/*z* of 113.0257) and a C_7_ fragment (C_7_H_8_O_3_, with an *m*/*z* of 139.0412). These results indicate a multistep degradation mechanism involving both ring-opening and oxidative mineralization processes, consistent with efficient BPA photodegradation upon prolonged photocatalytic treatment by CPE-PH–SO_3_® (Figure 5).^55,56^

**Figure 5:**
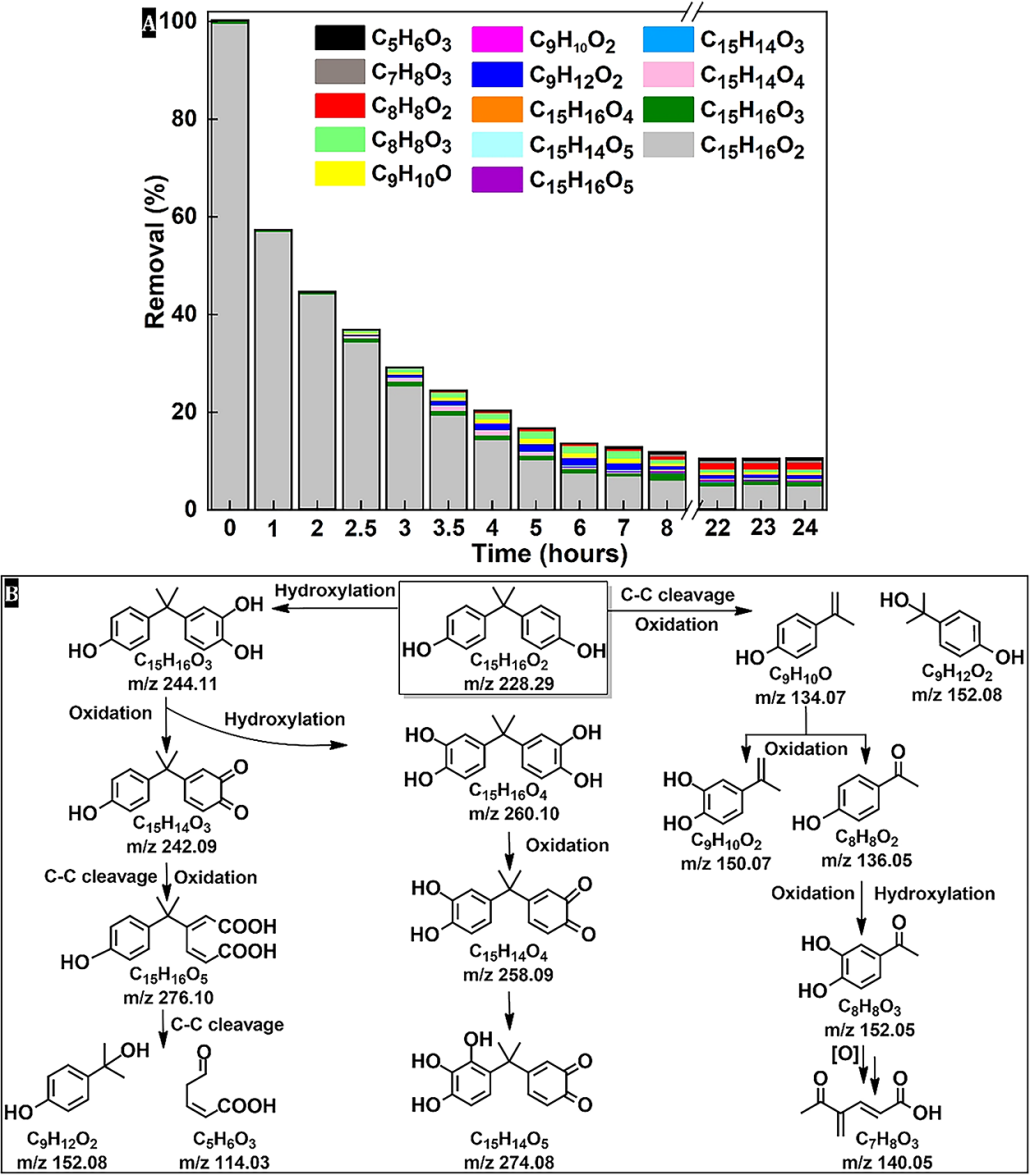
Removal efficiency of BPA from water and the relative contributions of the main BPA degradation intermediates in 24 h (A) and possible photodegradation pathway of BPA in the presence of CPE-PH–SO_3_® under ViS light irradiation (B) analyzed with DART-MS and LC-ToFMS. The *m*/*z* ratios given were obtained by DART-MS in negative ionization mode.

#### 3.4.4 Photochemical Stability and Durability of CPE-PH

After the 24 h BPA removal test, the stability of CPE-PH–SO_3_® was evaluated by ^13^C–CP/MAS NMR, UV–vis DRS, and SEM. The UV–vis DRS spectrum of the sample showed a slight decrease in the intensity of the absorption maximum (Figure 6a), indicating minimal electronic changes possibly due to partial surface contamination or minor photoinduced changes at the surface of the π-conjugated network, without significant backbone degradation. ^13^C–CP/MAS NMR revealed two additional peaks in the sample used (Figure 6b), a strong signal at ∼35 ppm indicating adsorbed BPA, and a new resonance at ∼170 ppm, likely originating from photooxidized BPA intermediates or minor oxidation of the conjugated backbone. Importantly, the key structural features of virgin CPE-PH–SO_3_® remained largely unchanged, suggesting that the π-conjugated network was preserved. SEM imaging also confirmed that the interconnected macroporous architecture remained intact. These results demonstrate that CPE-PH–SO_3_® retains both its structural integrity and optoelectronic functionality under prolonged photocatalytic conditions, which strengthens its potential for durable water treatment applications.

To further investigate the intrinsic photochemical stability of CPE-PH and to explain the observed differences in photocatalytic performance (Section 3.4.2), both CPE-PH–SO_3_® and CPE-PH-NMe_3_^+^ were subjected to accelerated artificial aging. These experiments were performed in the absence of BPA under continuous irradiation with visible light (λ > 420 nm) and continuous aeration (750 mL min^–1^ compressed air) for 24 h. This test environment was designed to simulate the most unfavorable operating conditions under which highly reactive ROS can begin to degrade the polymer photocatalyst’s backbone when no organic pollutant is present in the water. Under these conditions, CPE-PH-NMe_3_^+^ showed clear signs of significant photooxidative degradation, which correlates with its poor performance as a photocatalyst. The ^13^C CP/MAS NMR analysis of CPE-PH-NMe_3_^+^ showed new resonances appeared at δ 167 ppm and δ 194 ppm, corresponding to carboxylic acid^57^ and keto^58^ functionalities, respectively. Additional changes included a reduced signal intensity at δ 46 ppm (methyl groups of NMe_3_^+^), a slight attenuation at δ 71 ppm (CH_2_ in propyl chains), and the appearance of a new shoulder peak, probably due to the formation of terminal hydroxyl groups.^59^ Both samples showed reduced signal intensity in the range of δ 80–100 ppm (−C≡C−), in addition to the occurrence of a peak at δ 194 ppm. This is consistent with the formation of a diketo bridge by oxidative cleavage of the alkyne linker (Figures S14 and S15).^60^ In contrast, CPE-PH–SO_3_® showed only one new peak at δ 194 ppm, indicating a more localized and limited backbone oxidation. The total organic carbon (TOC) analysis confirmed these results. The initial TOC values at time 0 h (0.75 ppm for CPE-PH–SO_3_® and 1.27 ppm for CPE-PH-NMe_3_^+^) likely reflect trace organic contaminants (Table S6). After 22 h, the TOC values increased to 2.04 ppm for CPE-PH–SO_3_® and 3.99 ppm for CPE-PH-NMe_3_^+^, confirming higher susceptibility to self-oxidation under photocatalytic conditions for the cationic analogue. Overall, these results emphasize the superior photochemical robustness of CPE-PH–SO_3_®, which resists oxidative photodegradation even under harsh, substrate-free conditions, further supporting its selection as the most promising CPE-PH hydrogel for long-term photocatalytic water treatment.

**Figure 6:**
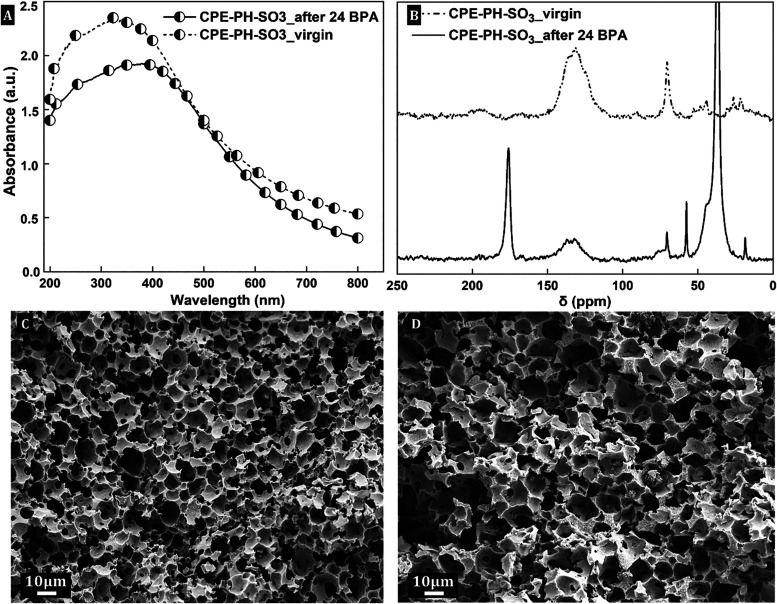
UV–vis DRS spectra (A), ^13^C–CP/MAS NMR spectra (B), and SEM images (C, D) of the CPE-PH–SO_3_® photocatalyst before and after the 24 h BPA removal test.

The same CPE-PH–SO_3_® monolith was reused in consecutive BPA removal runs to assess the operability information. The overall removal was maintained over two cycles without a detectable loss of performance (Figure S16). In the third run, however, magnetic stirring fragmented the HIPE monolith into fine powder, some of which was lost during workup. This mass loss ultimately resulted in a negligible photoactivity of CPE-PH–SO_3_®. Postcycling UV–Vis DRS showed no significant change in the absorption profile, confirming the high photochemical stability of the conjugated network under repeated irradiation and aeration. Although this bench-scale BPA photodegradation test did not achieve the high recycle/reuse number, it revealed a key hurdle, the mechanical fragility of CPE-PHs. This suggests a potential area for improvement by developing more robust and mechanically resistant high-porosity CPE hydrogels.

## 4 Conclusions

In this work, we have developed high-porosity conjugated polyelectrolyte hydrogels (CPE-PHs) using high internal phase emulsion (HIPE)-templated synthesis. CPE-PH hydrogels combine π-conjugation, ionic functionality, and permanent macroporosity in a single, cross-linked monolithic structure. Among the synthesized PHs, the anionic CPE-PH–SO_3_® exhibited exceptional dual-function performance in the removal of bisphenol A (BPA) from water by achieving ∼93% or ∼96% total removal through a synergistic combination of adsorption (∼71% or ∼50%) and visible light-driven photocatalysis (∼22% or ∼46%) in 8 or 24 h experiment. The fluorescence probe method confirmed the formation of hydroxyl (OH^•^) and superoxide anion O_2_^•^ ® radicals under visible-light irradiation, which correlates with the CPE-PH’s photocatalytic activity. A comparative analysis with the cationic analogue CPE-PH-NMe_3_^+^ revealed that it suffers from structural instability under oxidative conditions, resulting in limited photocatalytic performance. Stability studies using solid-state NMR, UV–vis-DRS, SEM and TOC analysis confirmed that CPE-PH–SO_3_® retained its structural integrity and optoelectronic functionality after prolonged photocatalytic operation (24 h), while CPE-PH-NMe_3_^+^ underwent significant oxidative degradation.

These results suggest that the high-porosity CPE-PHs hydrogels presented herein, in particular, the robust anionic CPE-PH–SO_3_®, represent a promising multifunctional polymer platform for advanced water remediation, enabling simultaneous adsorption and visible light-driven heterogeneous photocatalytic degradation under mild conditions. This dual functionality is in line with current efforts to intensify processes in water treatment, as the large surface area, water compatibility, and integrated light harvesting enable the use of smaller, more efficient reactor volumes while maintaining the same high contaminant removal efficiency. In addition, the synthetic modularity and tunable ionic functionalities of CPE-PH hydrogels enable the targeting of a broader range of emerging contaminants, including pharmaceutical residues and dyes, beyond BPA.

The main limitation of the CPE-PH hydrogels for water pollutant removal is photon transport through the monolith. The HIPE-derived macroporous architecture scatters visible light, slightly reducing the photocatalytic contribution compared to adsorption. The next step in addressing this issue is translucency engineering. This could be achieved by creating mesoporous structures within a CPE hydrogel network or using isorefractive HIPE organogels as templates. Another way to improve light penetration and thus photocatalytic efficiency is through reactor design by coating CPE-PH on light-permeable supports. These approaches should preserve the high sorption capacity and robustness of the CPE hydrogel network while increasing photon utilization and overall removal efficiency.

## Supplementary Material



## Data Availability

Data will be made available upon request.

## Notes

Details on preparation, elemental analyses, NMR, FTIR, UV–vis DRS, SEM, pictures, and adsorption measurements (PDF)
