# Supplementary material for: High-Porosity Conjugated Polyelectrolytes Synthesized via Sonogashira–Hagihara Coupling in Concentrated Emulsions: Robust Adsorptive–Photocatalytic Hydrogels for Water Pollutant Removal
Source: Macromolecules. 2026 Jan 6;59(2):901–12. doi: 10.1021/acs.macromol.5c02304 (PMC12854763; doi:10.1021/acs.macromol.5c02304)
Supplement: Supplementary file 1 [file ma5c02304_si_001.pdf]

Supplementary information

High-Porosity Conjugated Polyelectrolytes Synthesized via Sonogashira-Hagihara Coupling in Concentrated Emulsions: Robust Adsorptive–Photocatalytic Hydrogels for Water Pollutant Removal

Aleksander Saša Markovič[a,b], Siebe Lievens[c], Emeline Hanozin[c], Milica Velimirovic[c], Albin Pintar[b], Sebastijan Kovačič[a,b,*]

[a]Catalysis and Organic Synthesis research group, Faculty of Chemistry and Chemical Engineering, University of Maribor, Smetanova 17, SI-2000 Maribor, Slovenia

[b]Department of Inorganic Chemistry and Technology, National Institute of Chemistry, Hajdrihova 19, SI-1001 Ljubljana, Slovenia

[c]Flemisch Institute for Technological Research (VITO), Boeretang 200, 2400 Mol, Belgium

[*]Corresponding author. *E-mail address:* sebastijan.kovacic@um.si (S. Kovačič).

1. Materials and methods

2,5-dibromohydroquinone (Fluorochem), 1,3-propanesultone (Aaron chemicals), 3-bromopropane-N,N,N-trimethyl ammonium bromide (Aaron chemicals), sodium bromide (Sigma Aldrich), hydrogen peroxide (ABCR) 1,3,5-triethynyl benzene (Aaron chemicals), palladium acetate (Aaron chemicals), tetrakis(triphenyl phospine) palladium (Merck), sodium hydroxide (Merck), Pluronic F108 (Sigma Aldrich), dimethyl sulfoxide (Merck), petroleum benzine (Merck), ethanol (Merck), toluene (Merck) and p-Xylene (Merck) were used as received unless stated otherwise.

Methods

NMR spectroscopy

Solid state NMR measurements of prepared polymers were performed on a Bruker Avance 400 MHz spectrometer and measurements in the liquid phase were carried out on a Bruker Avance 400 MHz and 600 MHz spectrometers. Chemical shifts are given in ppm relative to a SiMe4 standard. NMR data were processed using MestReNova and Bruker TopSpin software.

FTIR spectroscopy

FTIR spectroscopy was performed on the dried grinded samples using a Perkin Elmer (Inc., Waltham, MA, USA) FTIR spectrometer with attenuated total reflection (ATR) in a 400-4000 cm^{-1} range at a resolution of 4 cm^{-1}. The presented spectra are an average of 16 consecutive measurements on Ge-crystal.

SEM microscopy

Scanning electron microscopy (SEM) images were collected on a JWS-7515, JEOL scanning electron microscope. The samples were attached to a carbon tab for better conductivity and afterwards a thin layer of Pt was sputtered on the sample's surface prior to scanning analysis.

Nitrogen physisorption

Nitrogen physisorption analysis was determined from the adsorption and desorption isotherms of N_2 at -196 °C using a Micromeritics TriStar II 3020 instrument. Prior to characterization, the samples were degassed under N_2 stream (purity 6.0) using a programmed bilevel heating, with the first heating stage at 90 °C for 60 min, followed by the second heating stage at 120 °C for 240 min. The heating rate was set to 10 °C min^{-1} for both heating stages. The specific surface area of the samples was calculated by applying the BET theory to the nitrogen adsorption data within the 0.06−0.30 p/p^0 range.

He-pycnometry

The polyHIPE skeletal densities (ρ_P) (an average of 10 consecutive measurements) were evaluated using a fully automated, highly precision helium pycnometry (Micromeritics, model AccuPyc II 1340). To prevent the influence of moisture and impurities on the measurements, the polyHIPEs were thoroughly dried and purged with nitrogen.

UV-Vis DR spectroscopy

UV-Vis DR spectroscopy was performed on a Perkin Elmer Lambda 650 UV-Vis spectrophotometer equipped with the accessory for powdered samples in order to record the UV-Vis diffuse reflectance spectra of the prepared materials. The background correction was performed with a white reflectance standard Spectralon® (range of 200−800 nm).

Cyclic voltammetry

Cyclic voltammograms were taken on potentiostat/galvanostat (Metrohm Autolab) at scan rate 100 mV/s and 25 °C in a three-electrode electrochemical cell. Electrolyte was 0.1 M tetrabutylammonium hexafluorophosphate (TBAPF6) in acetonitrile. To prepare the working electrode, a volume of 10 µL of catalyst-ethanol suspension (12.5 mg of catalyst in 2.5 mL of absolute ethanol (Sigma Aldrich, Germany)) was dropped onto the surface of the screen-printed DropSens electrode. Reference electrode was a calomel electrode (HANNA instruments, model HI5412) and a counter electrode was made of platinum.

DART-MS

To investigate the degradation mechanism of the developed photocatalyst and to identify potential compounds generated by the photocatalytic process, a non-targeted screening approach was applied. This non-targeted screening was executed using a high-resolution accurate mass Q Exactive Orbirtrap mass spectrometer (MS) from ThermoFisher, equipped with a Direct Analysis in Real Time (DART) ionization source from IonSense. The DART source was controlled via the DART-SVP software interface, while the mass spectrometer operations were managed using ExactiveTM software from ThermoFisher.

During the analysis, the liquid samples were held in front of the DART source with a glass capillary (VWR) without any sample preparation. The helium, which was heated to 400 °C and served as a carrier gas, came out of the source in an excited state. These excited helium species were used to ionize the molecules present in the samples, which were then introduced into the mass spectrometer. The MS was operated in negative ionization mode while the scan range was set between 50 and 750 m/z with a resolution of 140 000 and a scan rate of 3.7 scans per second. In addition, the MS was calibrated with ThermoFisher's PierceTM FlexMixTM calibration solution prior to imaging.

After performing the data acquisition, the obtained data was treated with an in-house developed workflow. This workflow included the usage of the FreeStyleTM 1.8 SP2 QF1 from ThermoFisher, an in-house build database containing the accurate mass of numerous chemical compounds, and a custom-build python script. Accurate masses obtained from the experiments were matched against the database to assign chemical identities. Unmatched compounds underwent manual assessment, and based on the accurate mass, a chemical formula was assigned.

Liquid Chromatography Coupled to Quadrupole Time of Flight Mass Spectrometry (LC-QToFMS)

Subsequent to the identification of potential compounds introduced by the photocatalysis and linked to bisphenol A, a confirmation was executed using a more extensive approach. The temporal pattern of bisphenol A and its generated derivatives were mapped using Liquid Chromatography (Elute+ UHPLC pump HPG1300, Bruker) coupled to Quadrupole Time of Flight Mass Spectrometry (timsTOF Pro 2 in TOF mode, Bruker). The LC instrument was equipped with a C18-BEH-phenyls column (2.1 mm × 100 mm, 1.7 µm) from Waters and held at 40 °C. The injection volume used was 5 µL, while the mobile phases (flow rate 0.4 mL/min) were (A) ultrapure water (ChromasolvTM LC-MS Ultra, Honeywell Chemicals) containing 2 mmol/L ammonium acetate (VWR) and (B) acetonitrile (Biosolve B.V.). The applied separation gradient started at 80% (A) and went to 5% (A) in 8 min. Afterwards, the gradient was kept for 2 min and went back to 80% (A) in 0.10 min. This last gradient was then held for 1.90 min. At the beginning of each acquisition, the mass spectrometer was calibrated using an in-house made sodium formate cluster solution. Therefore, 250 mL ultrapure water (ChromasolvTM LC-MS Ultra, Honeywell Chemicals) was mixed with 250 mL isopropanol (Honeywell Chemicals), 1 mL formic acid (Sepulco) and 5 mL sodium hydroxide (Sepulco) 1

N. Data treatment was performed using the Compass DataAnalysis Version 6.1 and TASQ Version 2024.1.3 software packages from Bruker.

Tests of photocatalytic activity of prepared polymer networks

All photocatalytic experiments were carried out in a batch slurry reactor (Lenz Laborglas, model LF60, 250 mL) made of glass, using water-dissolved bisphenol A (BPA, Aldrich) as a model pollutant and a 150 W halogen lamp (Philips) equipped with an UV cut-off filter at 410 nm. The photocatalytic oxidation_tests were performed at atmospheric pressure and 20 °C using a Julabo thermostat (model F25/ME). 250 mL of aqueous solution of BPA (c_0=10.0 mg/L) was mixed with the selected photocatalysts to form a suspension (c_{cat}=130 mg/L). The BPA/photocatalyst suspension was stirred at 500 rpm and purged with air at a rate of 45 L/h. Before illumination, the suspension was kept in the dark for 2 h to establish the sorption process equilibrium of BPA onto the catalyst surface. Samples were taken at different times in the reaction timespan and filtered using a 0.2 μm regenerated cellulose membrane filter.

The temporal BPA concentration in the withdrawn aqueous-phase samples was measured using a Shimadzu HPLC LC-40 instrument equipped with a 100 × 4.6 mm BDS Hypersil C18 (2.4 μm) column. The mobile phase was a mixture of methanol (Baker) and ultrapure water (70:30 vol ratio) with a flow rate of 0.5 mL/min. The column was thermostated at 30 °C and the autosampler at 25 °C. The detection of BPA was carried out with a PDA detector from 190 to 350 nm with a flow-through cell temperature of 40 °C.

2. Experimental: Synthesis and characterization of monomers, polymers and foams

2.1. Synthesis of monomers

2,5-bis(3-[N,N,N-trimethylamino]-1-oxapropyl)-1,4-dibromobenzene (M-NMe_3^+)

268 mg (1 mmol) of 2,5-dibromohydroquinone, 654 mg (2.5 mmol) of (3-Bromopropyl)trimethylammonium bromide and 100 mg (1.25 mmol) of NaOH were dissolved in 4 mL of ethanol. The reaction mixture was refluxed at 80 ºC for 48 h. After, the mixture was allowed to cool to room temperature after which the product crystalized out of solution, the product was collected by filtration. The crude product was dissolved in a hot mixture of MeCN and water (20:1), the solution was hot filtered, after which the product crystallized out, to yield 600 mg (95.5%) of pure product.

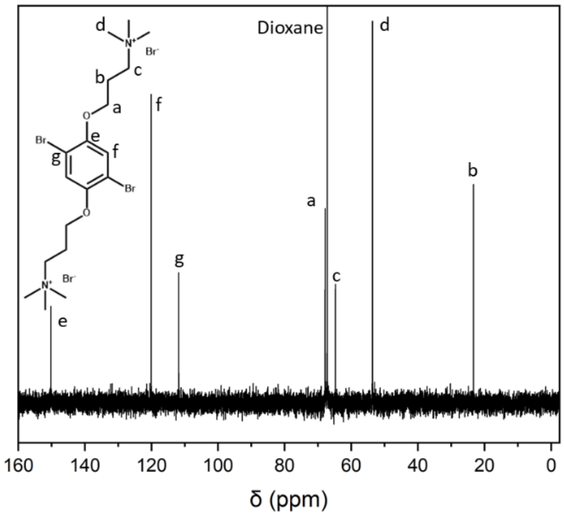

Figure S1. 13C NMR spectra of M-NMe3+.

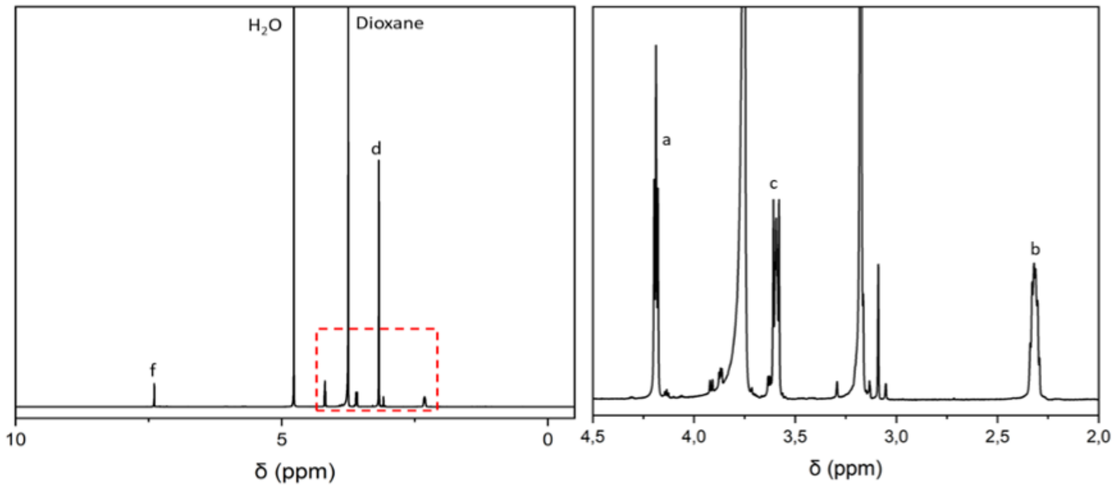

Figure S2. 1H NMR spectra of M-NMe_3^+.

^{13}C NMR (151 MHz, D_2O) δ 150.12, 120.05, 111.80, 67.84, 64.71, 53.62, 23.32. **^{1}H NMR** (600 MHz, D_2O) δ 7.34 (s, 2H), 4.12 (t, J = 5.7 Hz, 4H), 3.55 – 3.49 (m, 4H), 3.10 (s, 18H), 2.24 (dq, J = 11.3, 5.6 Hz, 4H).

Synthesis of 1,4-dibromo-2,5-bis(3-sulfonatopropoxy)benzene disodium salt (M-SO_3^-)

The monomer was prepared according to a modified procedure described in literature [1]. 536 mg (2 mmol) of 2,5-dibromohydroquinone, 610 mg (5 mmol) of 1,3-propanesultone and 200 mg (5 mmol) of NaOH were dissolved in 15 mL of ethanol. The reaction mixture was refluxed at 80 °C overnight. After, the mixture was allowed to cool to room temperature after which the product crystalized out of solution, the product was collected by filtration and washed by EtOH to obtain the pure product and dried to constant mass, to yield 1,11 g (100%) of pure product.

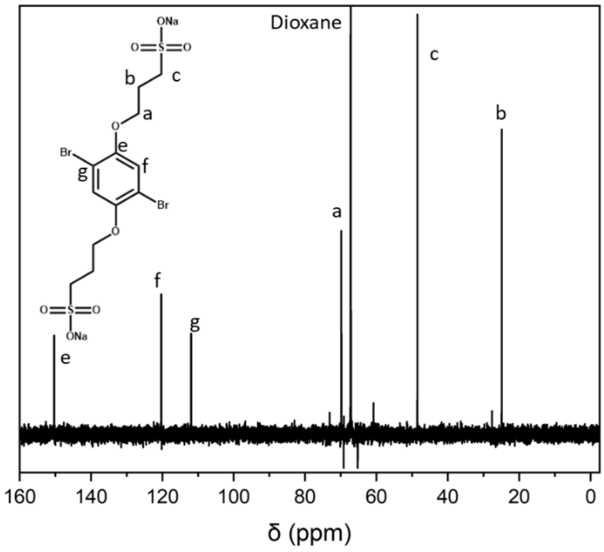

Figure S3. 13C NMR spectra of M-SO_3^-.

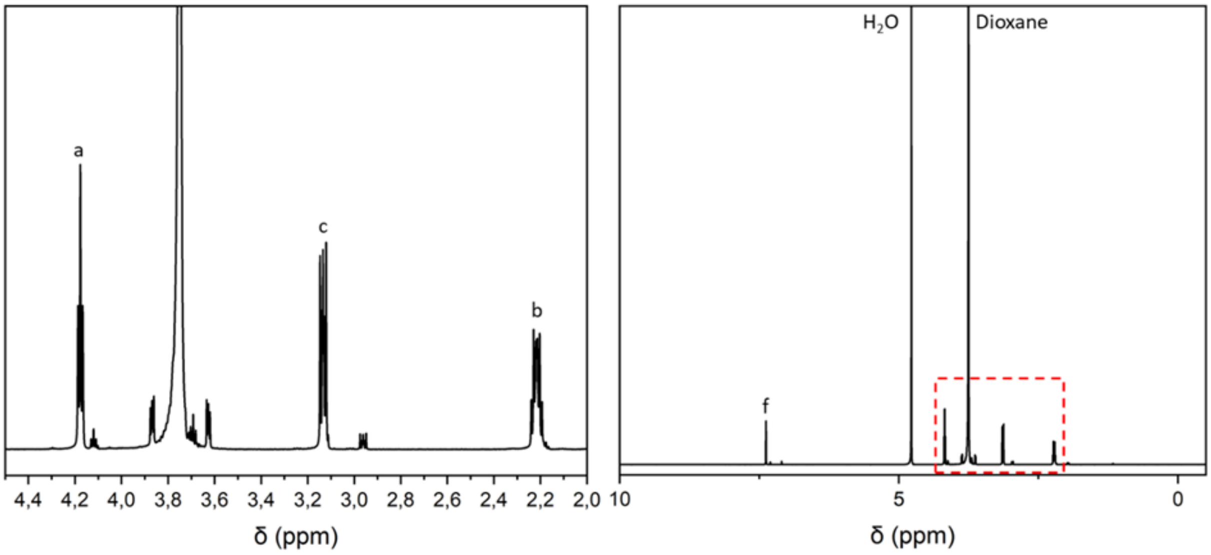

Figure S4. 1H NMR spectra of M-SO_3^-.

^{13}C NMR (151 MHz, D_2O) δ 150.28, 120.27, 111.89, 69.83, 48.52, 24.87. **^{1}H NMR** (600 MHz, DMSO) δ 7.30 (s, 2H), 4.05 (t, *J* = 6.4 Hz, 4H), 2.66 – 2.61 (p, 4H), 2.03 – 1.95 (p, 4H).

Table S1. Solubility of reactants in various solvents.

	DMSO	NMP	DMF	GVL	Cyrene	EG	NMP/H_2O
**M-NMe_3^+**	✓*	x	x	x	x	✓	✓
**M-SO_3^-**	x	x	x	x	x	✓*	✓
**TEB**	✓	✓	✓	✓	✓	✓	✓
**DABCO**	✓	✓	✓	✓	✓	✓	✓
**NEt_3**	✓	✓	✓	✓	✓	✓	✓
**$Pd(PPh_3)_4$**	✓	✓	✓	✓	✓	x	x
**$Pd(OAc)_2$**	✓	✓	✓	✓	✓	✓	✓
**$PdCl_2$**	✓	✓	✓	/	/	✓	✓

*At elevated temperature.

Table S2. Yields of solution polymerization in DMSO with different sources of Pd.

	PAE-SO_3	PAE-NMe_3
**$Pd(PPh_3)_4$**	62.1%	62.5%
**$Pd(OAc)_2$**	26.7%	43.1%
**$PdCl_2$**	x	x

2.2. Synthesis of nonporous polymer monoliths

Polymer monoliths based on 2,5-bis(3-[N,N,N-trimethylamino]-1-oxapropyl)-1,4-dibromobenzene (P-NMe_3^+)

41.4 mg (0.035 mmol) Pd(PPh3)$_2$ was added to a vial, 220 mg (0.35mmol) of cationic monomer, 79 mg (0.53 mmol) of 1,3,5-triethynyl benzene and 235 mg of DABCO were dissolved in 1.5 mL DMSO. The suspension was transferred to a preheated oven at 80 ^oC and was left to polymerize overnight. The prepared monolith was first purified by Soxhlet extraction using a 1:1 mixture of ethanol:water for 24 h, remaining impurities were removed by $scCO_2$ drying, to yield a polymer monolith with a yield of polymerization of 62.5%.

Polymer monoliths based on 1,4-dibromo-2,5-bis(3-sulfonatopropoxy)benzene disodium salt (P-SO_3^-)

41.4 mg (0.035 mmol) Pd(PPh3)$_2$ was added to a vial, 195 mg (0.35mmol) of anionic monomer, 79 mg (0.53 mmol) of 1,3,5-triethynyl benzene and 235 mg of DABCO were dissolved in 1.5 mL DMSO. The suspension was transferred to a preheated oven at 80 ^oC and was left to polymerize overnight. The prepared monolith was first purified by Soxhlet extraction using a

1:1 mixture of ethanol:water for 24 h, remaining impurities were removed by $scCO_2$ drying, to yield a polymer monolith with a yield of polymerization of 62.1%.

2.3. Synthesis of polymer foams

CPE-PH-SO_3^-

41.4 mg (0.035 mmol) $Pd(PPh3)_2$ was added to a vial, 217 mg of pluronic F-108 (10 wt.%), 195 mg (0.35 mmol) of anionic monomer, 79 mg (0.53 mmol) of 1,3,5-triethynyl benzene and 235 mg of DABCO were dissolved in 1.5 mL DMSO. To this mixtures 4.5 mL petroleum benzine was added dropwise as internal phase (IP), after all of IP was added the emulsion was left to stir for another 15 min. Then the emulsion was transferred to a preheated oven at 80 ^oC and was left to polymerize overnight. The prepared monolith was first purified by Soxhlet extraction using a 1:1 mixture of ethanol:water for 24 h, remaining impurities were removed by $scCO_2$ drying, to yield a macroporous polymer foam with a yield of polymerization of 64%.

CPE-PH-NMe_3^+

41.4 mg (0.035 mmol) $Pd(PPh3)_2$ was added to a vial, 219 mg of pluronic F-108 (10 wt.%), 220 mg (0.35 mmol) of cationic monomer, 79 mg (0.53 mmol) of 1,3,5-triethynyl benzene and 235 mg of DABCO were dissolved in 1.5 mL DMSO. To this mixtures 4.5 mL petroleum benzine was added dropwise as internal phase (IP), after all of IP was added the emulsion was left to stir for another 15 min. Then the emulsion was transferred to a preheated oven at 80 ^oC and was left to polymerize overnight. The prepared monolith was first purified by Soxhlet extraction using a 1:1 mixture of ethanol:water for 24 h, remaining impurities were removed by $scCO_2$ drying, to yield a macroporous polymer foam with a yield of polymerization of 80.6%.

2.4. Oxo addition to alkyne and polyacetylene synthesis

24 mg of $Pd(OAc)_2$ was dissolved in ethylene glycol, then 527 µL of phenylacetylene was added to the mixture. It was left to react at 80 °C for 24 h, after that the mixture was allowed to cool to room temperature, it was then diluted by 50 mL of demineralized water and precipitate formed which was extracted with EA, the collected organic phases were washed with brine and water, then dried over $MgSO_4$. The tar like product stuck to the bottom of the flask was diluted in acetone, for both organic phases, the solvent was removed under reduced pressure and the crude mixture were analyzed by NMR.

NMR spectra suggest the presence of polyacetylene in both samples, as seen from Figures S5 and S6, where the broad peak at 7.45 ppm is contributed to the polymer [2]. Whereas in the fraction isolated by acetone, we can more profoundly see product 2b [3], with peaks between 1 and 6 ppm.

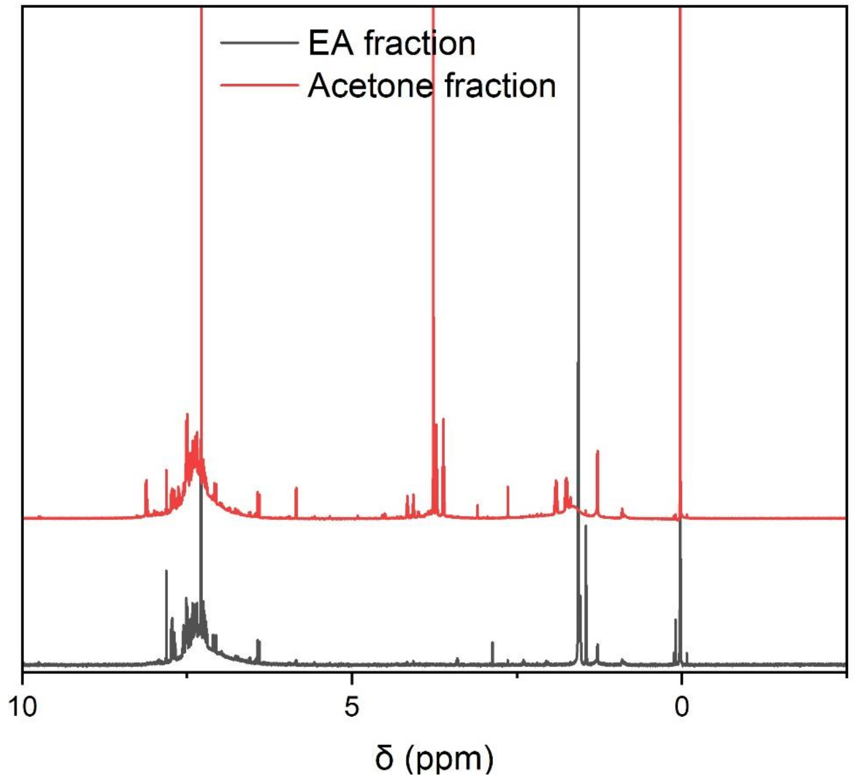

Figure S5. Sample fraction extracted by EA.

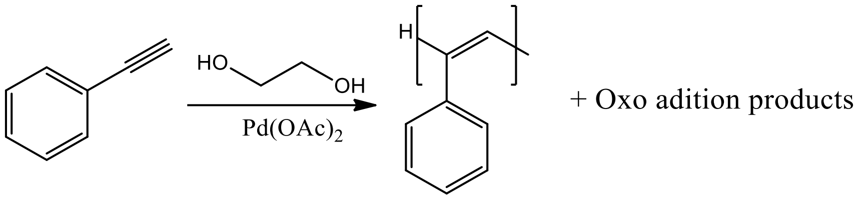

Figure S6. Oxo addition to alkyne and polyacetylene synthesis.

3. Characterization of polymer foams

Gel content

An onset of gelation during polymerization was determined by a vial inversion method by noting a time when the reacting liquid stopped flowing. The chemical gel formation was further confirmed by refluxing selected samples in DMSO and determining the gel fraction of the polyHIPEs. Model CPE-PH cubes were refluxed in DMSO at 150 °C for 24 hours. The extraction was stopped after 12 h, the samples were purged with pure warm solvent, then dried and weighed.

Table S3. Elemental analysis.

Found					
	C %	H %	N %	S %	mmol pendant group/ g polymer
PAE-SO_3	55.9	5.2	2.9	3.0	0.94
PAE-NMe_3	65.0	5.6	3.7	0	2.64
Theoretical					
	C %	H %	N %	S %	
PAE-SO_3	48.5	3.4	0	13.0	4.06
PAE-NMe_3	55.1	6.2	4.9	0	3.50

Equation for mmol pendant group/ g polymer:

$$\frac{n_x}{1g} = \frac{w_x\%}{100 \cdot M_x}$$

n_x - mmol of pendant group

$w_x\%$ - mass percent of element X

M_x - molar weight of element X

FTIR spectroscopy analysis

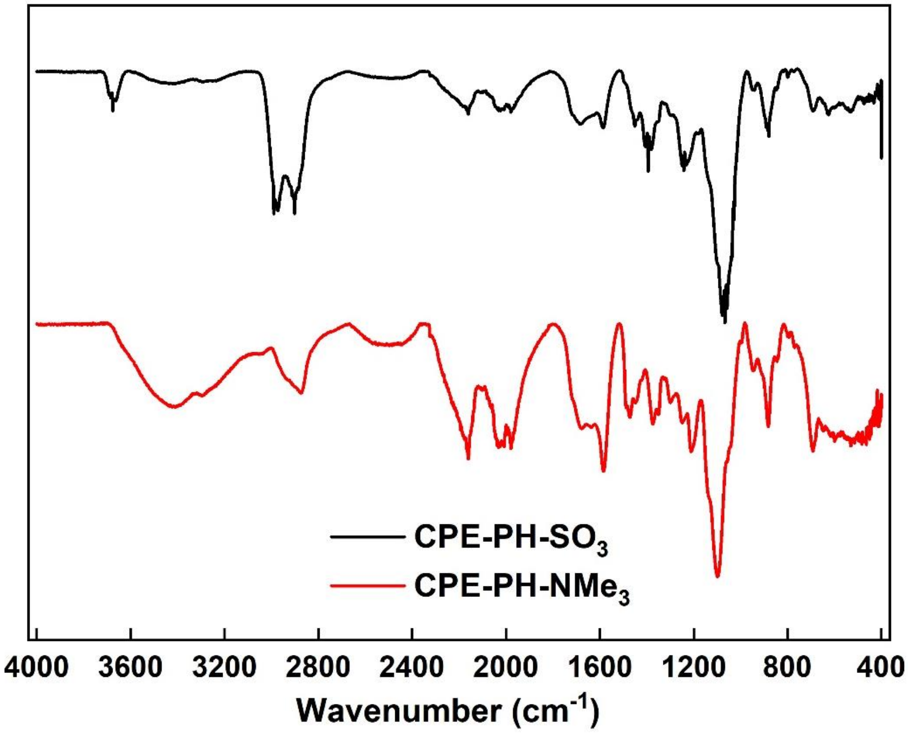

Figure S7. FTIR spectra of CPE-PH hydrogels.

^{13}C CP/MAS NMR spectroscopy

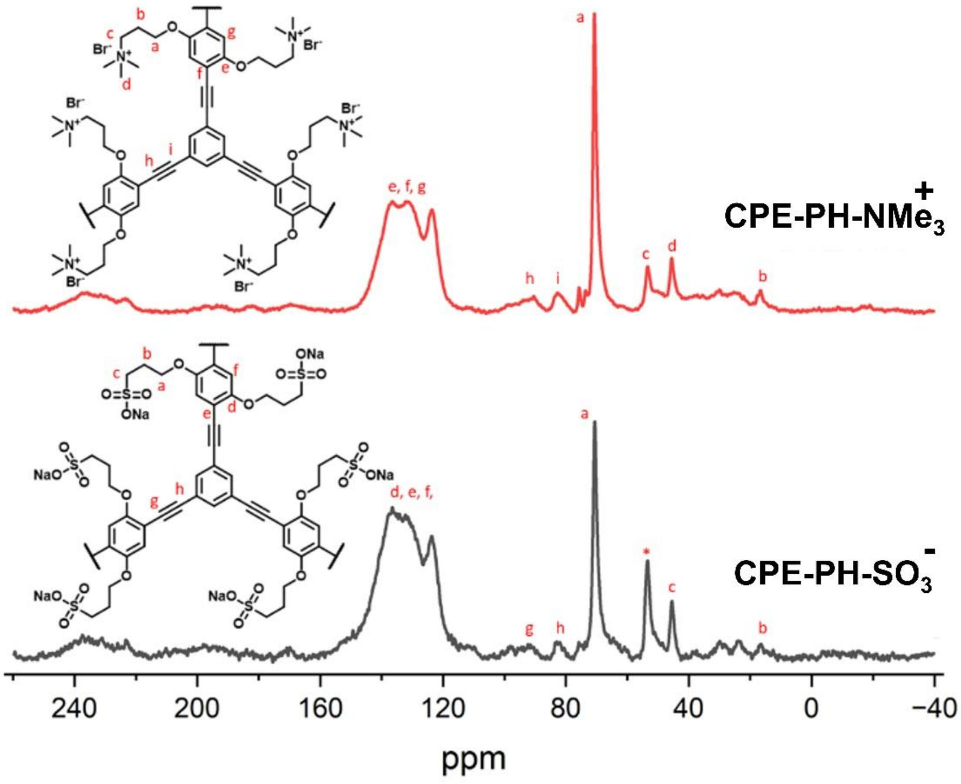

Figure S8. ^{13}C CP/MAS of CPE-PH.

HIPE formulation

Sample	PAE-SO_3	PAE-NME_3
Organic Internal Phase, wt.%		
DMSO (Solvent)	29.82	29.68
SO_3 (anionic monomer)	3.52	--
NMe_3 (cationic monomer)	--	3.96
1,3,5-triethynylbenzene (crosslinker)	1.43	1.42
F108 (surfactant)	3.92	3.90
Pd $(PPh_3)_4$ (catalyst)	0.74	0.74
DABCO	4.22	4.20
Total	43.44	43.67
Organic Internal Phase, wt.%		
Petrolum benzin	56.56	56.33
Total	56.56	56.33

Table S4. Average pore size, density, porosity and specific surface area measurements.

**Samples**	**PAE-NMe$_3$**	**PAE-SO$_3$**
**$d_v \pm \sigma$ [µm]^a**	25±6	38±18
**$d_W \pm \sigma$ [µm]^b**	5±1	9±3
**ρ_{PH} [g·cm^{-3}]^c**	0.061	0.056
**ρ_P [g·cm^{-3}]^d**	1.7950±0.0174	1.7088±0.0252
**P [%]^e**	96	97
**SSA [m^2·g^{-1}]^f**	299	354

[a]Average void diameter, [b]average window diameter, [c]dry polyHIPE density, [d]polymer skeletal density determined by He-pycnometry, [e]porosities of monoliths, [f]specific surface area.

Porosity equation:

$$P = (1 - \frac{\rho_{PH}}{\rho_P}) \cdot 100\%$$

Table S5. Water uptake.

**Samples**	**PAE-NMe$_3$**	**PAE-SO$_3$**
**Uptake [g/g]^a**	17.1	25.0

[a]Mass of soaked monolith after 2 h (plateau), divided by initial mass.

Fluorescent probe techniques

Coumarin test for $OH^{\bullet}$ radicals

The formation of $OH^{\bullet}$ radicals was measured by using fluorescence probe method with coumarin (COUM, Alfa Aesar, Haverhill, MA, USA, 98%, p.a) as a probe molecule. Upon reaction with radicals different hydroxycoumarins can be formed, where only 7-hydroxycoumarin (7-OHC) is suitable to be followed by fluorescence probe method. 10 mg of a photo-catalyst was suspended in 50 mL of 1.4 mM COUM aqueous solution. The suspension was stirred in the dark for 20 min before illumination with a visible LED lamp (Schott, model KL 1600 LED, Germany). The samples were then analyzed by recording the fluorescence signal of the generated 7-OHC using a Perkin Elmer UV–Vis fluorescence spectrophotometer (model LS 55, USA). The scanning speed was 200 nm min^{-1} and the wavelength of the excitation light was set to 338 nm. The excitation and the emission slits were both set to the width of 10 nm.

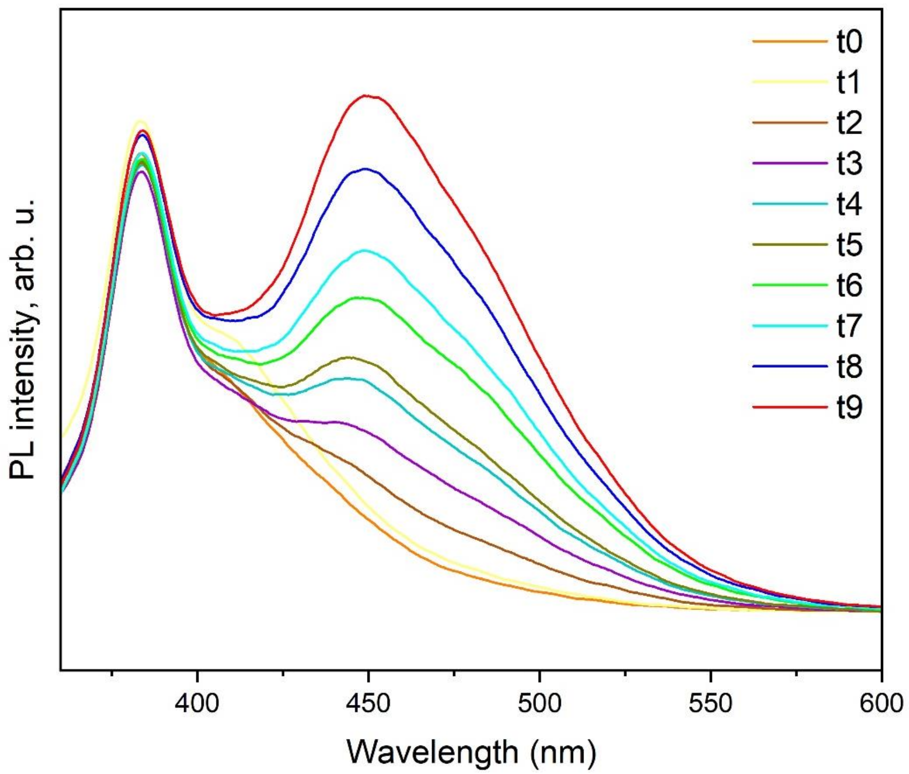

Figure S9. PL spectra - $OH^{\bullet}$ radical test.

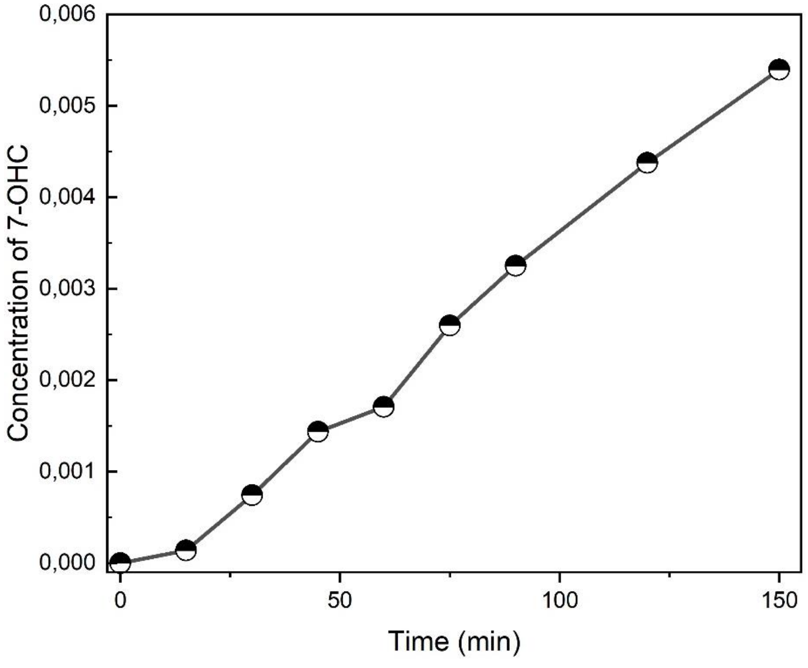

Figure S10. Concentration of 7-OHC - $OH^{\bullet}$ radical test.

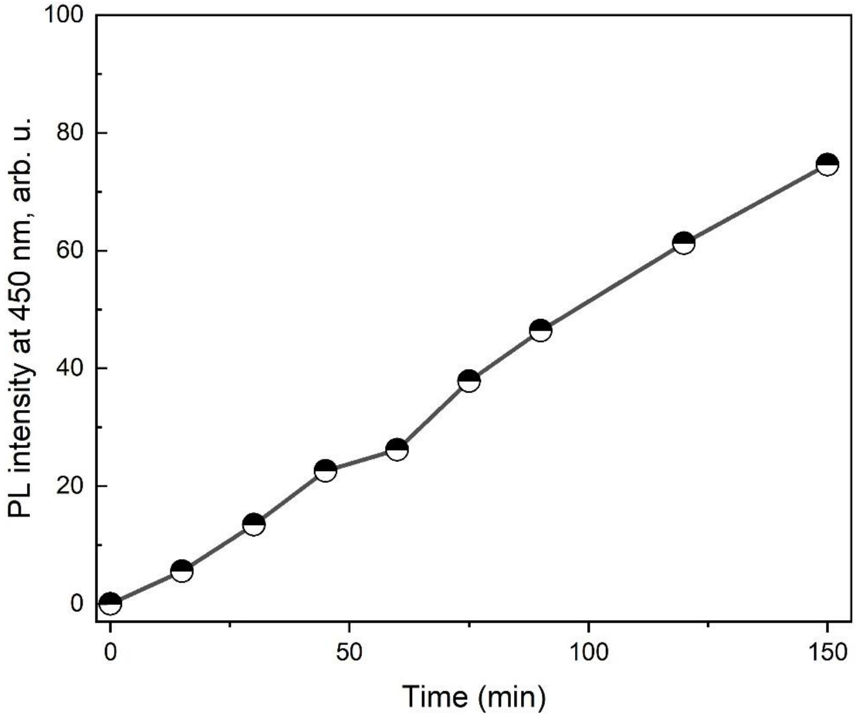

Figure S11. PL intensity at 450 nm - OH$^{\bullet}$ radical test.

ABTS test for $e^-/O_2^{\cdot -}$

17.2 mg of 2,2'-azino-bis(3-ethylbenzothiazoline-6-sulphonic acid (ABTS, Sigma-Aldrich, Germany) and 3.3 mg of potassium peroxodisulfate ($K_2S_2O_8$, Sigma-Aldrich, Germany) were dissolved in 5 mL of ultrapure water and stirred for 16 h to obtain ABTS radical cation ($ABTS^{\cdot +}$) solution. After that 1 mL of $ABTS^{\cdot +}$ solution was diluted in 50 mL of ultrapure water, into which 10 mg of a catalyst were suspended. The suspension was stirred in the dark for 30 min before illumination with a visible LED lamp (Schott, model KL 1600 LED, Germany). Aqueous-phase samples were withdrawn after 0, 15, 30, 45, 60, 75 and 90 min of light illumination and immediately filtered through a 0.2 nm membrane filter. To follow the reduction of $ABTS^{\cdot +}$a Perkin

Elmer Lambda 45 UV–Vis spectrophotometer was employed to record UV–Vis absorbance spectra of the ABTS solutions.

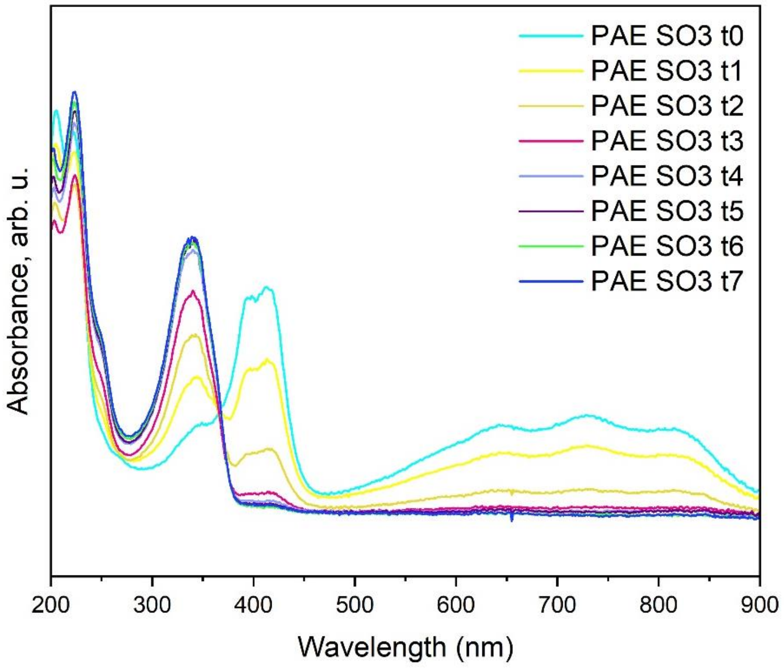

Figure S12. UV-Vis spectra - ABTS$^{\cdot+}$ reduction.

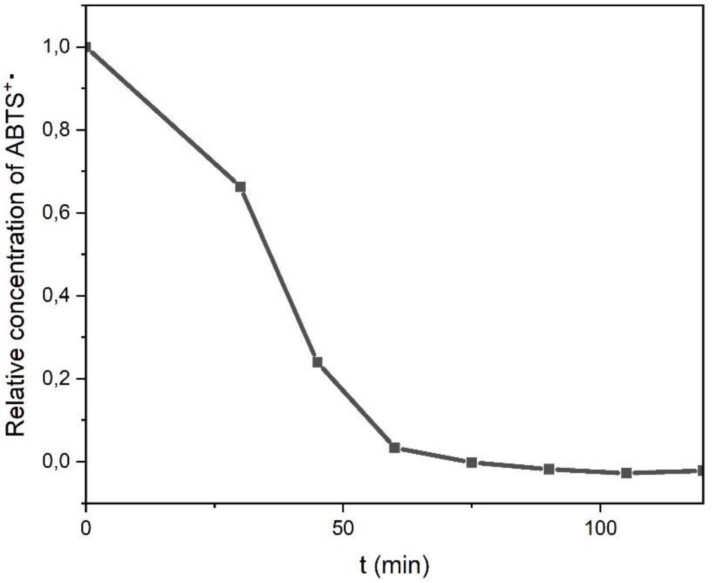

Figure S13. Reduction of ABTS^{+} to ABTS.

Photochemical stability tests

A batch reactor was covered with aluminium foil and all ambient lights were turned off, after 250 mL of demineralized H_2O (without the presence of BPA) and 65 mg of an appropriate catalyst was added. The solution was aerated at 750 mL/min for 24 h. After 24 h the samples were filtered from the solution and dried in a fume hood. ssNMR measurements were conducted on dry samples.

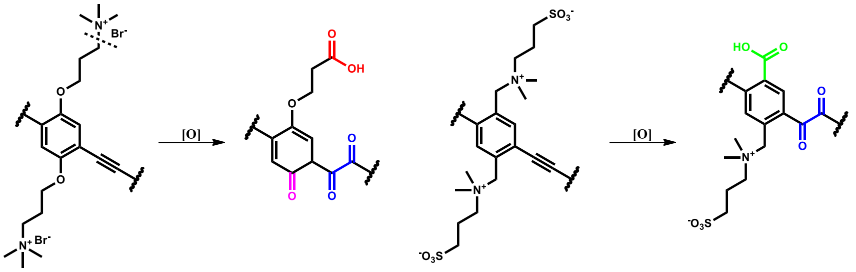

Figure S14. Plausible defects in polymer catalyst after irradiation.

Table S6. TOC measurements - 24 h photo stability tests.

Sample	T_0 [ppm]	T_1 [ppm]	T_2 [ppm]
**PAE-SO$_3$**	0.75	2.09	2.44
**PAE-NMe$_3$**	1.27	2.22	3.99

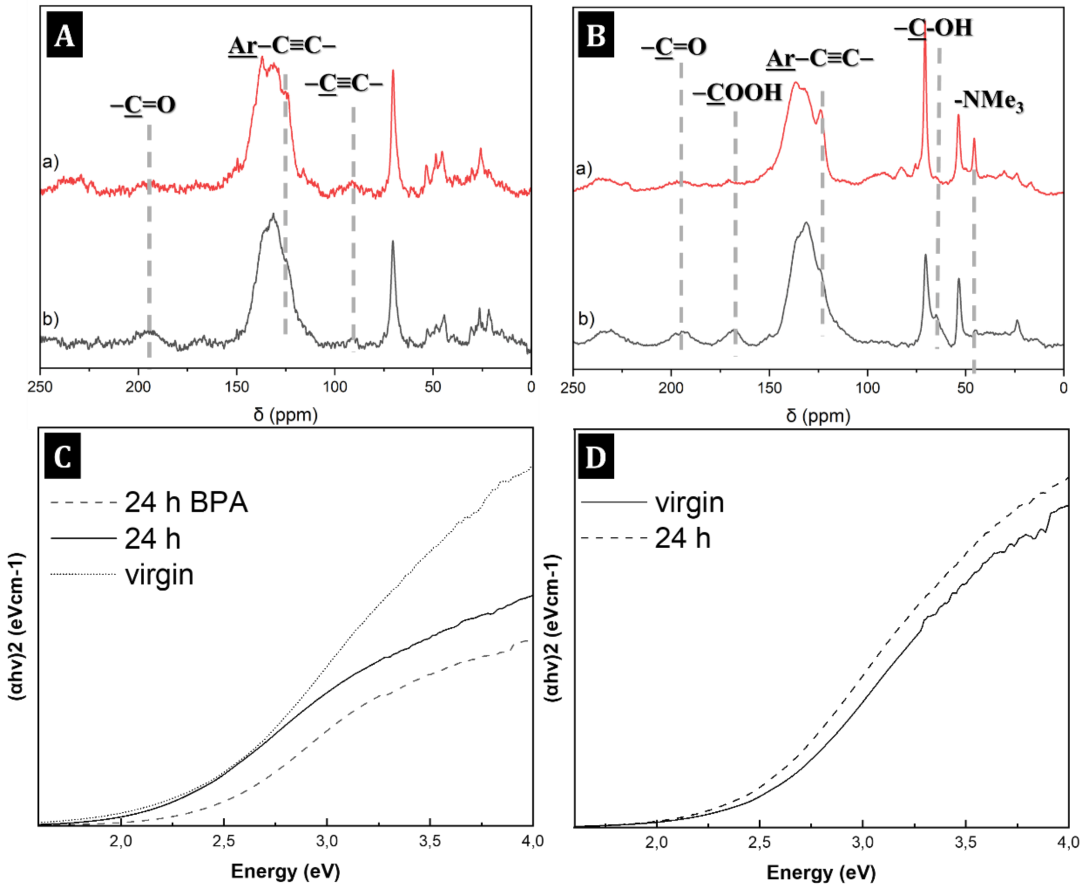

Figure S15. CP/MAS NMR spectra before and after photochemical stability test for CPE-PH-SO_3^- **(A)** and CPE-PH-NMe_3^+ **(B),** Tauc plot before and after photochemical stability test for CPE-PH-SO_3^- **(C)** and CPE-PH-NMe_3^+ **(D)**.

Reusability test

A batch reactor was covered with aluminum foil and all ambient lights were turned off, after 250 mL of 10 ppm BPA solution. The reusability tests were conducted in the same manner as 8 hour runs. Three consecutive runs were performed, after each run the catalyst was washed with water and acetone, then the catalyst was dried in a vacuum oven for 16 h at 80 ºC. Aliquots at different times of reaction were analyzed by HPLC.

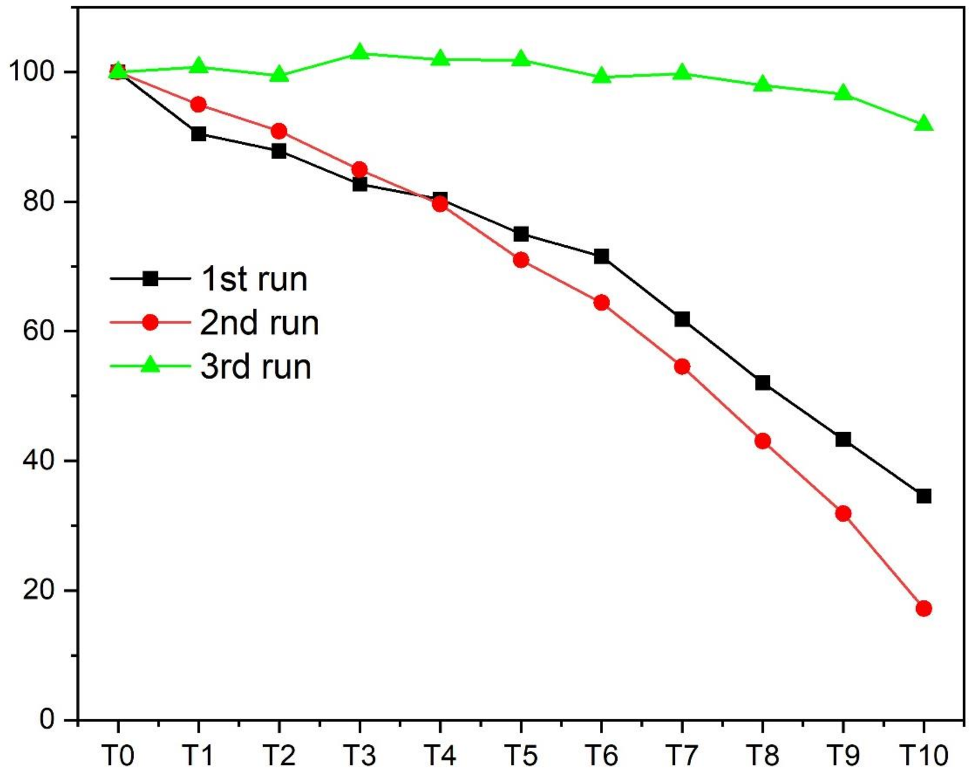

Figure S16. Recyclability tests of CPE-PH-SO_3^-

References

(1) Jang, G.; Kim, J.; Kim, D.; Lee, T. S. Synthesis of Triphenylamine-Containing Conjugated Polyelectrolyte and Fabrication of Fluorescence Color-Changeable, Paper-Based Sensor Strips for Biothiol Detection. *Polym. Chem.* **2015**, *6* (5), 714–720. https://doi.org/10.1039/C4PY01201A.

(2) Grünwald, A.; Heinemann, F. W.; Munz, D. Oxidative Addition of Water, Alcohols, and Amines in Palladium Catalysis. *Angew. Chem. Int. Ed.* **2020**, *59* (47), 21088–21095. https://doi.org/10.1002/anie.202008350.

(3) Tan, Y.-F.; Yang, D.; Yang, Y.-H.; Lv, J.-F.; Zong, L.-X.; Guan, Z.; He, Y.-H. Electricity-Driven 1,4-Alkoxydimerization of Alkenes *via* Radical–Polar Crossover. *Green Chem.* **2023**, *25* (22), 9388–9393. https://doi.org/10.1039/D3GC02701E.
